# Updated component analysis method for naturally occurring sophorolipids from *Starmerella bombicola*

**DOI:** 10.1007/s00253-024-13138-x

**Published:** 2024-04-12

**Authors:** Yosuke Kobayashi, Qiushi Li, Kazunori Ushimaru, Makoto Hirota, Tomotake Morita, Tokuma Fukuoka

**Affiliations:** 1 Allied Carbon Solutions Co., Ltd., 847-1 Ozuwa, Numazu, Shizuoka 410-0873 Japan; 2 Research Institute for Sustainable Chemistry, National Institute of Advanced Industrial Science and Technology (AIST), Tsukuba Central 5-2, 1-1-1, Higashi, Tsukuba, Ibaraki 305-8565 Japan

**Keywords:** Biosurfactant, Component analysis, Glycolipid, LC–MS analysis, Sophorolipid, *Starmerella bombicola*

## Abstract

**Abstract:**

Sophorolipids (SLs) are promising glycolipid biosurfactants as they are easily produced and functional. SLs from microorganisms are comprised of mixtures of multiple derivatives that have different structures and properties, including well-known acidic and lactonic SL (ASLs and LSLs, respectively). In this study, we established a method for analyzing all SL derivatives in the products of *Starmerella bombicola*, a typical SL-producing yeast. Detailed component analyses of *S. bombicola* products were carried out using reversed-phase high-performance liquid chromatography and mass spectrometry. Methanol was used as the eluent as it is a good solvent for all SL derivatives. With this approach, it was possible to not only quantify the ratio of the main components of ASL, LSL, and SL glycerides but also confirm trace components such as SL mono-glyceride and bola-form SL (sophorose at both ends); notably, this is the first time these components have been isolated and identified successfully in naturally occurring SLs. In addition, our results revealed a novel SL derivative in which a fatty acid is bonded in series to the ASL, which had not been reported previously. Using the present analysis method, it was possible to easily track compositional changes in the SL components during culture. Our results showed that LSL and ASL are produced initially and that SL glycerides accumulate from the middle stage during the fermentation process.

**Key points:**

• *An easy and detailed component analysis method for sophorolipids (SLs) is introduced.*

• *Multiple SL derivatives were identified different from known SLs.*

• *A novel hydrophobic acidic SL was isolated and characterized.*

**Graphical Abstract:**

**Figure:**
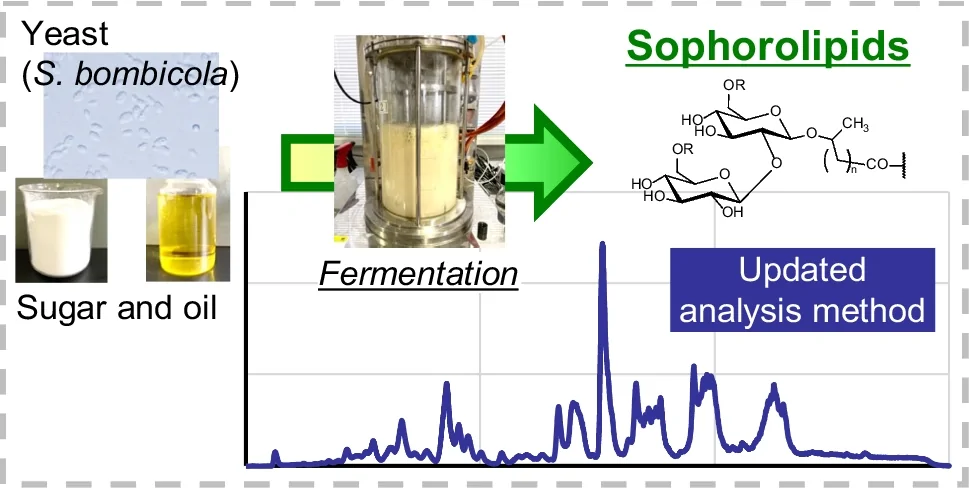

**Supplementary Information:**

The online version contains supplementary material available at 10.1007/s00253-024-13138-x.

## Introduction

**Fig. 1:**
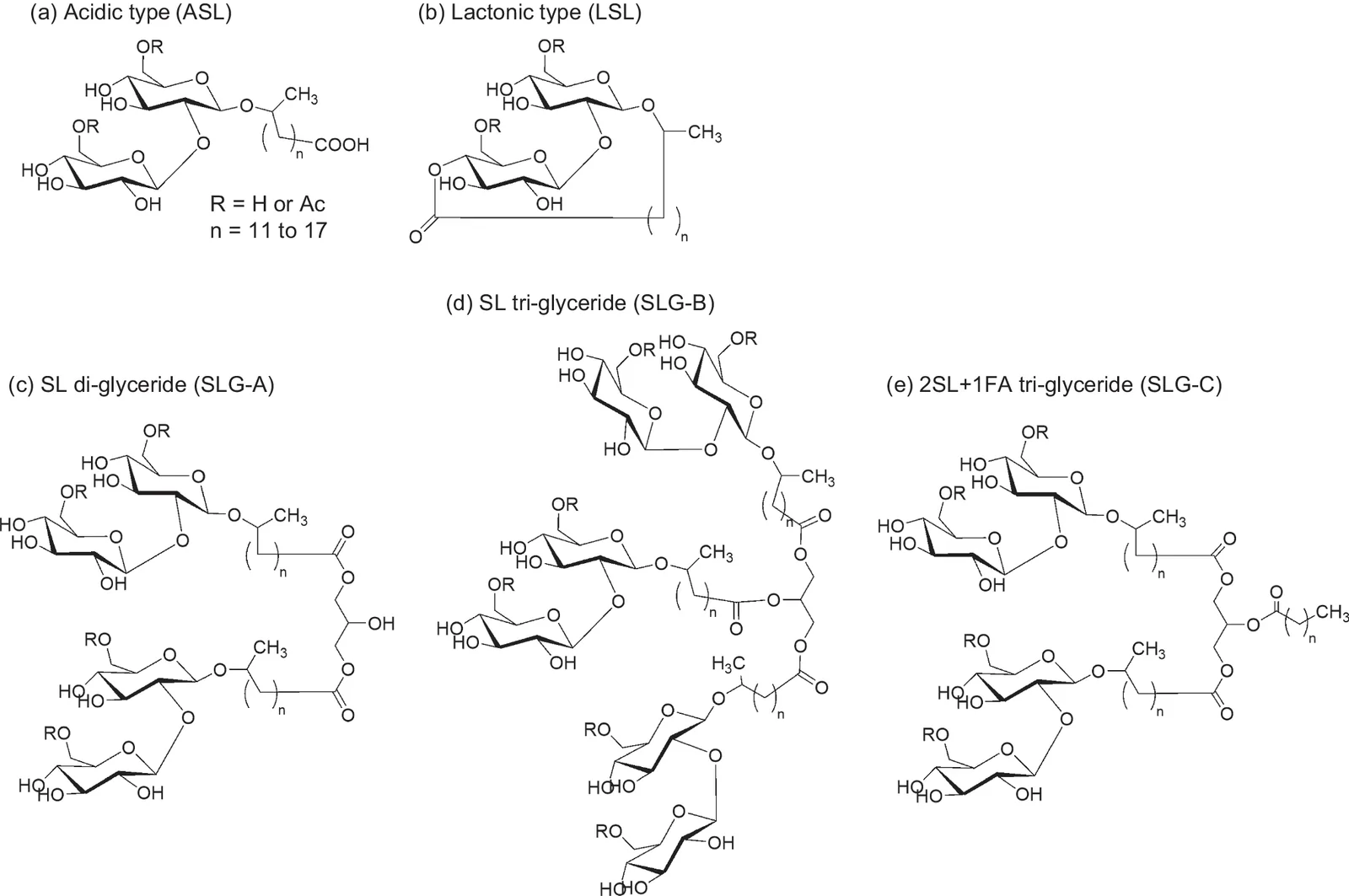
Chemical structures of well-known sophorolipid (SL) derivatives (**a**) Acidic sophorolipid (ASL), (**b**) lactonic sophorolipid (LSL), (**c**) sophorolipid di-glyceride (SLG-A), (**d**) sophorolipid tri-glyceride (SLG-B), and (**e**) 2-O-fatty acyl-1,3-di-O-sophorolipid glycerol (SLG-C: 2SL + 1FA tri-glyceride)

Sophorolipids (SLs, Fig. 1), one of the most commercialized glycolipid-type biosurfactants are amphiphilic glycolipids produced in abundance from biomass resources such as vegetable oils and sugars by bacteria, fungi, and yeasts (Ma et al. 2020; Li et al. 2020; Wongsirichot et al. 2021; Dierickx et al. 2022; Qazi et al. 2022). SLs are well known in academia and industry due to their high productivity and stability on a large scale. SLs can be produced at > 300 g/L using fed-batch culture techniques, and it is possible to increase productivity and recovery significantly using semi-continuous fermentation, a technique that combines fed-batch and separation processes (Dolman et al. 2017; Zhang et al. 2018). Recently, it has been reported that the total production of SLs can exceed 1,000 g/L of the working culture medium (Wang et al. 2020). SLs are produced commercially as biodegradable low-foaming surfactants with both high detergency and low cytotoxicity (Hirata et al. 2009a). In addition, the anticancer (Miceli et al. 2022), antimicrobial (Shu et al. 2021; Cho et al. 2022), and biofilm inhibition (Díaz De Rienzo et al. 2015; Haque et al. 2016; Nguyen et al. 2020) properties of SLs are well-known. As such, SLs are expected to apply in the food, agriculture, cosmetics, pharmaceutical, and medical industries (Dierickx et al. 2022; Pal et al. 2023).

*Starmerella bombicola*, a typical SL-producing yeast, produces a mixture of lactonic and acidic forms of SL (Fig. 1). The lactonic form of SL (LSL) is the main component in *S. bombicola* products and is considered to be the final product of SL biosynthesis. The acidic form of SL (ASL) is presumed to be produced as an intermediate during LSL biosynthesis or when the produced LSL undergoes hydrolysis. LSL tends to be a hydrophobic nonionic surfactant, whereas ASL is a hydrophilic water-soluble anionic surfactant. The critical micelle concentration (CMC) of ASL is more than tenfold higher than that of LSL, and the surface tension lowering activity of LSL is also significantly higher than that of ASL (Imura et al. 2013).

Interestingly, LSL and ASL compounds, which have completely different properties, can coexist in the same microbial product. There are reports of synergistic effects of LSL and ASL; for example, some mixtures show a lower CMC (Hirata et al. 2009b) and improved miscibility (Imura et al. 2014). On the other hand, the content ratio of these compounds in the microbial product varies depending on the culture conditions. Furthermore, their content ratios change easily even when in storage, as LSL is replaced by ASL through hydrolysis. Therefore, to expand the applications of SL products, we believe that it is important to accurately quantify the ratio of these components in SL products and to tightly control the composition and quality.

In our previous study, we reported that all SL components in an *S. bombicola* product can be analyzed without leaking by selecting an appropriate solvent (methanol) and combining normal- and reversed-phase chromatography (Kobayashi et al. 2023). With this approach, we found that there is a non-negligible amount of unknown glycolipids, in addition to conventional LSLs and ASLs. We isolated and characterized them as SL-glycerides (SLGs), i.e., glycerol esterified by multiple SLs, and propose that they are “the third type of naturally occurring SLs” after LSLs and ASLs. We have high expectations regarding the significance and synergistic effects of these SLGs as water-soluble nonionic surfactants that exhibit intermediate properties between LSL and ASL, in *S. bombicola* products. In this study, we aimed to establish an SL component analysis method for finding new SL derivatives. By conducting a detailed component analysis of *S. bombicola* products using the proposed method, we confirmed that naturally occurring SLs contain several previously overlooked components, including a new unreported SL derivative. We were also able to track changes in component ratios due to differences in the culture conditions and changes in the components over time during fermentation.

## Materials and methods

### Materials and microorganisms

All reagents and solvents were commercially available and used as received. Rapeseed oil (J-Oil Mills, Inc., Tokyo, Japan) and rice bran oil (Tsuno Food Industrial Co., Ltd., Wakayama, Japan) were used as received. *Starmerella bombicola* NBRC10243 (National Institute of Technology and Evaluation Biological Resource Center [NBRC], Chiba, Japan) was used for SL production. *Bacillus subtilis* NBRC3134 (gram-positive bacterium, NBRC), *Staphylococcus aureus* NBRC13276 (gram-positive bacterium, NBRC), and *Escherichia coli* NBRC3301 (gram-negative bacterium, NBRC) were used for antimicrobial activity assay of the SL derivatives.

### Microbial production of SLs using *Starmerella bombicola*

An *S. bombicola* pre-culture was prepared by inoculating cells cultured for 2 days at 30 °C in 100 mL of yeast extract–peptone–dextrose medium that contained 20 g/L glucose, 20 g/L peptone, and 10 g/L yeast extract. Two bottles of pre-culture were transferred to a 5-L jar fermenter containing 2 L of the main culture medium, which comprised 100 g/L glucose, 50 g/L rapeseed oil or rice bran oil, 10 g/L peptone, and 12 g/L yeast extract, for 3 days of cultivation. The fermenter (TSC-A5L; Takasugi Mfg. Co. Ltd., Tokyo, Japan) was operated at 25 °C and 800 rpm, with 1 vvm of airflow and with the pH controlled above 3 with NaOH. Sampling of the culture medium was performed at 0, 6, 12, 24, 48, and 72 h after the start of fermentation to track SL production. After the incubation period, a highly viscous precipitate containing the SLs was collected by heating at 60 °C for 1 h. The residual yeast cells were removed by centrifugation, and the viscous supernatant obtained was washed and dried.

### Component analysis of microbial products containing SLs from *Starmerella bombicola*

Thin-layer chromatography (TLC) of the compounds containing SLs was performed using anthrone-sulfate as a coloring reagent in the same manner as previously reported (Kobayashi et al. 2023). Normal-phase TLC (TLC silica gel 60 F254 glass plates; Merck, Darmstadt, Germany) was performed with a solvent system consisting of chloroform, methanol, and 12% ammonia solution (80:20:2, vol/vol/vol), to focus on detecting lower polar glycolipids such as LSLs. Reversed-phase TLC (TLC silica gel 60 RP-18 F254S glass plates; Merck) was conducted using a methanol and water (95:5, vol/vol) solvent system, to focus on detecting higher polar glycolipids such as ASLs.

High-performance liquid chromatography (HPLC) and liquid chromatography-mass spectrometry (LC–MS) analyses were carried out as previously reported with minor modification (Kobayashi et al. 2023). HPLC analysis was performed using an Ultimate 3000 HPLC system (Thermo Fisher Scientific, Waltham, MA, USA). The system was equipped with a C18 silica gel column (InertSustain C18 HP, 3 μm, 2.1 × 150 mm; GL Sciences, Inc., Tokyo, Japan), an InertSustain C18 guard column for UHPLC (3 μm, 2.1 × 100 mm; GL Sciences), and a charged aerosol detector (CAD) (Corona Veo; Thermo Fisher Scientific) using gradient elution of methanol and water containing 5 mM ammonium formate solvent systems (70:30 for 3 min, 70:30 to 100:0 for 15 min, 100:0 for 7 min, and 70:30 for 5 min) at a flow rate of 0.3 mL/min. SLs were quantified using standard curves generated from purified reference samples. When directly analyzing SLs in the culture medium, the collected culture media were diluted with 70% methanol containing 5 mM ammonium formate that was centrifuged to remove yeast cells, filtered, and subjected to HPLC analysis.

Liquid chromatography-mass spectrometry (LC–MS) analysis was carried out using the HPLC system described above, which was connected to a mass spectrometer equipped with an electrospray ionization source (Exactive Plus; Thermo Fisher Scientific). The instrument was operated in negative-ion electrospray mode, and the spectral range from 200 to 4,000 m/z was evaluated using Xcalibur software (ver. 3.0.63; Thermo Fisher Scientific).

### Isolation and purification of unknown glycolipids

Unknown glycolipids were isolated and purified using two-step silica gel column chromatography as previously reported with minor modification (Kobayashi et al. 2023). In the first step, normal-phase silica gel column chromatography was performed to separate LSLs and lower polar glycolipids from ASLs and higher polar glycolipids. The concentrated product containing the SLs was then dispersed in a small volume of chloroform and placed in a silica gel column (Wako-gel C-200; Fujifilm Wako Pure Chemical Co., Osaka, Japan). Purification was carried out using gradient elution of chloroform and acetone solvent systems (80:20 to 0:100, vol/vol). Residues adsorbed on the silica gel were eluted completely using methanol.

In the second step of the process, the collected fractions containing the target unknown glycolipids were dissolved in methanol, placed in a C18 silica gel column (Cosmosil 140C18-OPN; Nacalai Tesque, Inc., Kyoto, Japan), and purified using gradient elution of methanol and water solvents (70:30 to 100:0, vol/vol). Each fraction was subjected to reversed-phase TLC, and single fractions were collected and concentrated.

### Measurement of unknown glycolipid structures

The structures of the purified glycolipids dissolved in CD_3_OD or CDCl_3_ were determined using proton (^1^H) and carbon (^13^C) nuclear magnetic resonance (NMR). Two-dimensional analyses were carried out, including ^1^H–^1^H correlation spectroscopy (COSY), heteronuclear single quantum correlation (HSQC), and heteronuclear multiple bond coherence (HMBC), using a 400-MHz spectrometer (AVANCE III; Bruker, Bremen, Germany).

### Surface tension of SL derivatives

The surface tension of the purified SL derivative aqueous solutions was measured using the pendant drop method in the same manner as previously reported (Kobayashi et al. 2023).

### Antimicrobial activity testing

The antimicrobial activity assay against *B. subtilis*, *S. aureus*, and *E. coli* of the SL derivatives based on a modified broth dilution method (Reller et al. 2009) was performed in the same manner as previously reported (Kobayashi et al. 2023).

## Results

### Solubility of SLs in various solvents

The motivation for updating our SL component analysis method using HPLC was the presence of undissolved SL components in the analysis sample obtained using the conventional method. Therefore, we evaluated the solubility of the SL components in common solvents. Our results are summarized in Table 1. The well-known LSL and ASL showed opposite solubility in polar and nonpolar solvents, as expected. SLGs, the third SL type identified in our previous study, also showed differences in solubility that depended on the specific structure. Additionally, although LSL alone is insoluble in water, *S. bombicola* products that were mixtures of LSL and ASL were easily dispersed in water. Taken together, the results of the above solubility test showed that LSL, ASL, and mixtures thereof dissolved completely in methanol, thus showing great promise for use as an HPLC eluent.

**Table 1:** Solubility of sophorolipids and related compounds in various solvents^a^

	hexane	chloroform	ethyl acetate	acetone	acetonitrile	methanol	water
ASL (Fig. 1a)	-	-	-	-	-	+	+
LSL (Fig. 1b)	-	+	+	+	+	+	-
SL di-glyceride (SLG-A: Fig. 1c)	-	-	-	+	-	+	+
SL tri-glyceride (SLG-B: Fig. 1d)	-	-	-	+	-	+	+
2SL + 1FA tri-glyceride (SLG-C: Fig. 1e)	-	-	-	+	-	+	-
Compound X (bola-form: Fig. 3a)	-	-	-	-	-	+	+
Compound Y (SL mono-glyceride: Fig. 3b)	-	-	-	+	+	+	+
Compound Z (Fig. 3c)	-	+	-	+	-	+	-
*S. bombicola* products (SL mixtures)	-	-	-	± ^b^	-	+	+
olive oil	+	+	+	+	-	-	-
oleic acid	+	+	+	+	+	+	-
glucose	-	-	-	-	-	-	+

^a^ The solubility of each sample was adjusted to 10 mg/mL and checked visually. + : soluble or transparent, -: insoluble or cloudy

^b^ Solubility varied depending on composition of the sample

### Component analysis of S. bombicola products using reversed-phase HPLC and LC–MS

**Fig. 2:**
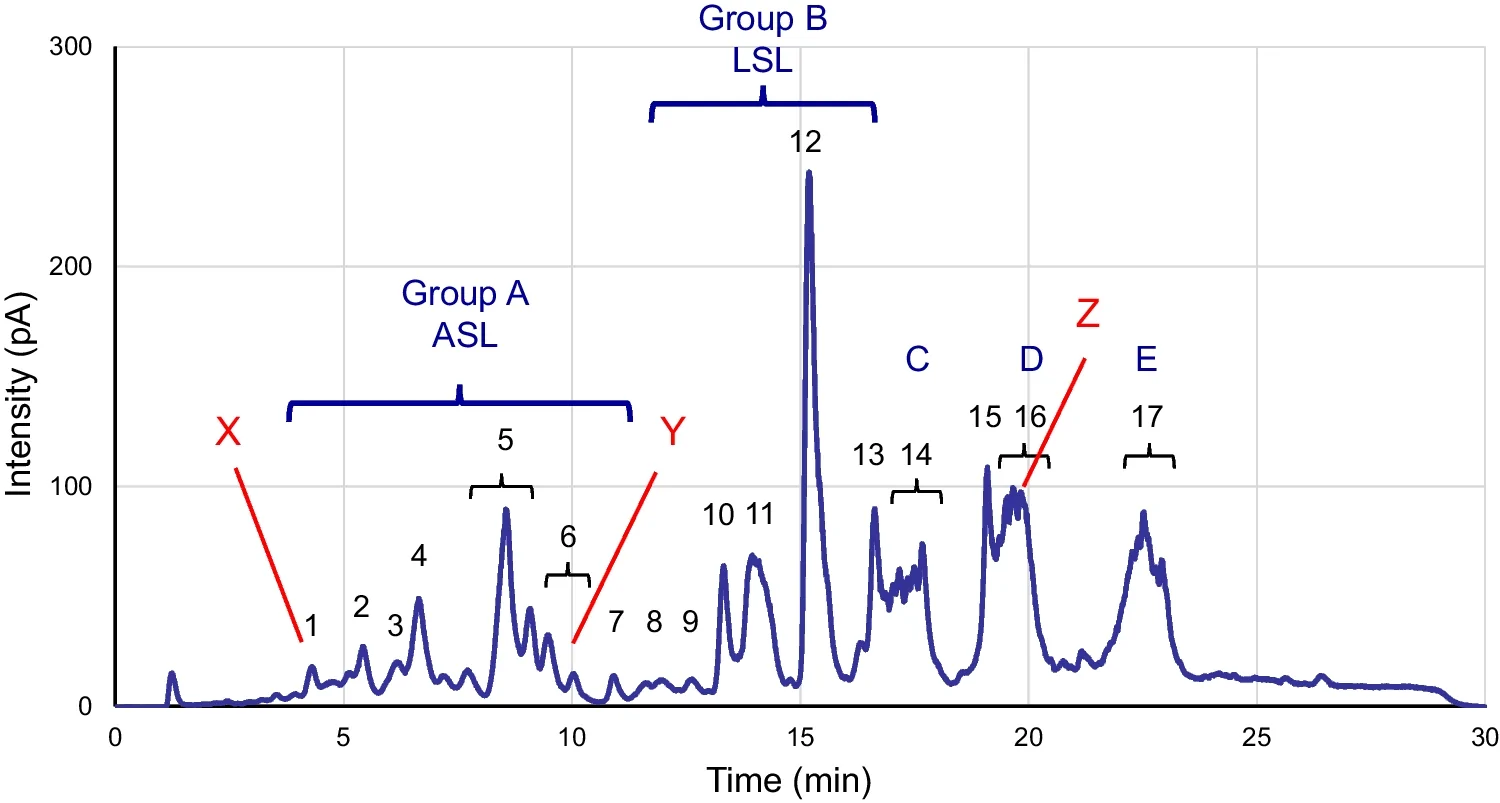
Charged aerosol detector high-performance liquid chromatography (CAD-HPLC) analysis of the collected microbial products of *S. bombicola* by jar fermentation from rapeseed oil using a C18 silica gel column and methanol/water eluent

Based on the above results, we attempted to analyze all SL components in *S. bombicola* products using reversed-phase HPLC in a methanol solvent system. Figure 2 shows an HPLC chart of *S. bombicola* products obtained from rapeseed oil by jar fermentation, as described in the experimental section. The peaks were assigned to ASL (group A in Fig. 2) or LSL (group B in Fig. 2) by analyzing the purified ASL and LSL samples. We previously reported that the three large peaks (peaks C–E in Fig. 2), eluted in the latter half, correspond to SL di- and tri-glycerides (Kobayashi et al. 2023). We further focused on three peaks containing unknown compounds (peaks X at retention time [r.t.] around 4.3 min, Y at r.t. around 9–10 min, and Z at r.t. around 19–20 min in Fig. 2) by comparing them with our LC–MS analysis results (Table 2).

**Table 2:** Peak list on HPLC chart of a *S. bombicola* product

No	r.t. (min)	m/z	Compound
1	4.30	621.4	ASL (Ac0 C18:1)
		661.4	ASL (Ac1 C18:2)
		1029.5	Unknown (Compound X)
2	5.42	663.4	ASL (Ac1 C18:1)
3	6.22	679.4	ASL (Ac2 C16:0)
		703.4	ASL (Ac2 C18:2)
4	6.62	663.4	ASL (Ac1 C18:1)
		703.4	ASL (Ac2 C18:2)
5	8.08–9.29	705.4	ASL (Ac2 C18:1)
6	9.30–10.56	779.4	Unknown (Compound Y)
7	10.90	707.4	ASL (Ac2 C18:0)
8	11.72	603.3	LSL (Ac0 C18:0)
		619.3	LSL (Ac1 C16:0)
		643.3	LSL (Ac1 C18:2)
9	12.59	643.3	LSL (Ac1 C18:2)
10	13.34	645.3	LSL (Ac1 C18:1)
11	13.95	645.4	LSL (Ac1 C18:1)
		661.3	LSL (Ac2 C16:0)
		685.3	LSL (Ac2 C18:2)
12	15.20	687.4	LSL (Ac2 C18:1)
13	16.61	689.4	LSL (Ac2 C18:0)
14	16.95–18.32	1467.8	SL di-glyceride (SLG-A: Fig. 1c)
15	19.10	281.2	Oleic acid
16	19.29–20.56	985.6	Unknown (Compound Z)
		2156.1	SL tri-glyceride (SLG-B: Fig. 1d)
17	21.58–23.78	1730.0	SL2 + FA1 tri-glyceride (SLG-C: Fig. 1e)

Table 2 shows that the mass of compound X [M-H]^–^ (m/z) was 1,029.5 in fraction No.1; this is different from any of the known values for LSL, ASL, or SL di- and tri-glycerides. The structure estimated from the molecular weight value of compound X is an SL derivative in which ASL is esterified to another disaccharide (probably sophorose). This compound is most likely “bora-form SL”, synthesized recently by culturing genetically modified microorganisms (Van Bogaert et al. 2016; Van Renterghem et al. 2018).

Other newly identified compounds included compound Y [M-H]^–^ (m/z), with a mass of 779.4 in fraction No.6, and compound Z [M-H]^–^ (m/z) with a mass of 985.6 in fraction No.16 (Table 2). Based on the structures estimated from the molecular weight values of compounds Y and Z, Y is an SL mono-glyceride, in which one ASL is esterified to glycerol, whereas Z is an SL derivative in which another fatty acid is esterified to ASL. To our knowledge, compound Z is a newly identified SL derivative; however, further analysis is required to determine its structure as this compound has many possible isomers.

### Isolation and identification of compounds X–Z

**Fig. 3:**
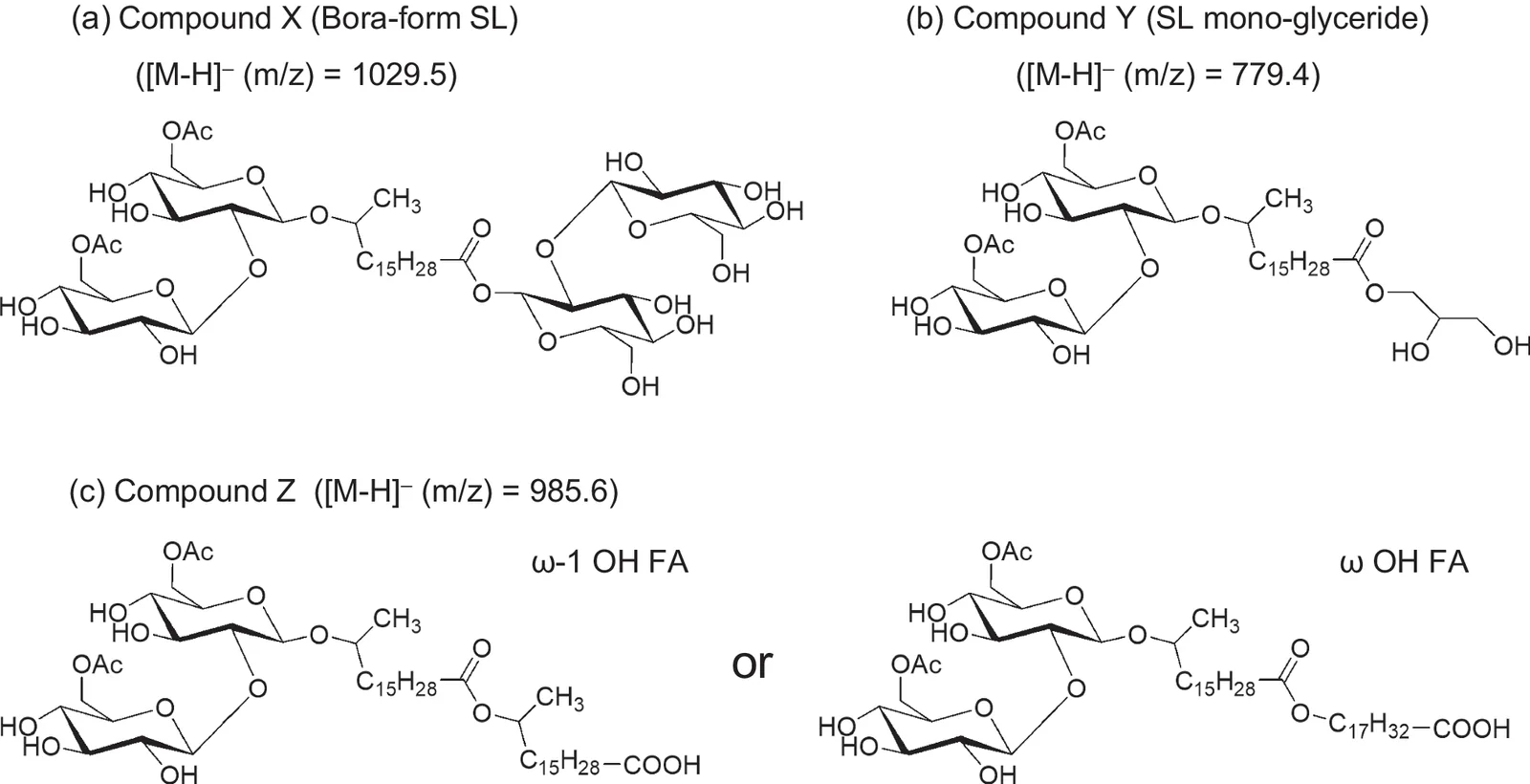
Putative chemical structures of the main components of three compounds newly found in *S. bombicola* products by CAD-HPLC and liquid chromatography- mass spectrometry (LC–MS) analysis. (**a**) Compound X (bola-form sophorolipid), (**b**) compound Y (sophorolipid mono-glyceride), and (**c**) compound Z

The three compounds X–Z were isolated by careful fractionation using C18 silica gel column chromatography. Compounds X and Y were trace components in the *S. bombicola* product. They could not be isolated by normal-phase silica gel column chromatography because they could not be separated from ASL. Therefore, we first separated LSL from the *S. bombicola* product using normal-phase silica gel column chromatography and recovered the remaining SL components, including ASL and other SL derivatives. Next, the remaining SL components were fractionated sequentially and precisely using reversed-phase C18 silica gel column chromatography. The results of LC–MS and NMR analyses of the isolated and purified compounds X and Y are shown in the Supplementary Information (Figs. S1–S9 and Table S1, S2). As expected, the estimated X and Y main components were bola-form SL and SL mono-glyceride, respectively (Fig. 3).

In contrast, the peak of compound Z completely overlapped that of SL tri-glyceride (SLG-B) on the reversed-phase HPLC chart (Fig. 2); however, these compounds can be separated. First, compound Z including LSL was separated from the *S. bombicola* product using normal-phase silica gel column chromatography. Compound Z was then separated from LSL and isolated using reversed-phase C18 silica gel column chromatography (Fig. S10 in the Supplementary Information).

**Fig. 4:**
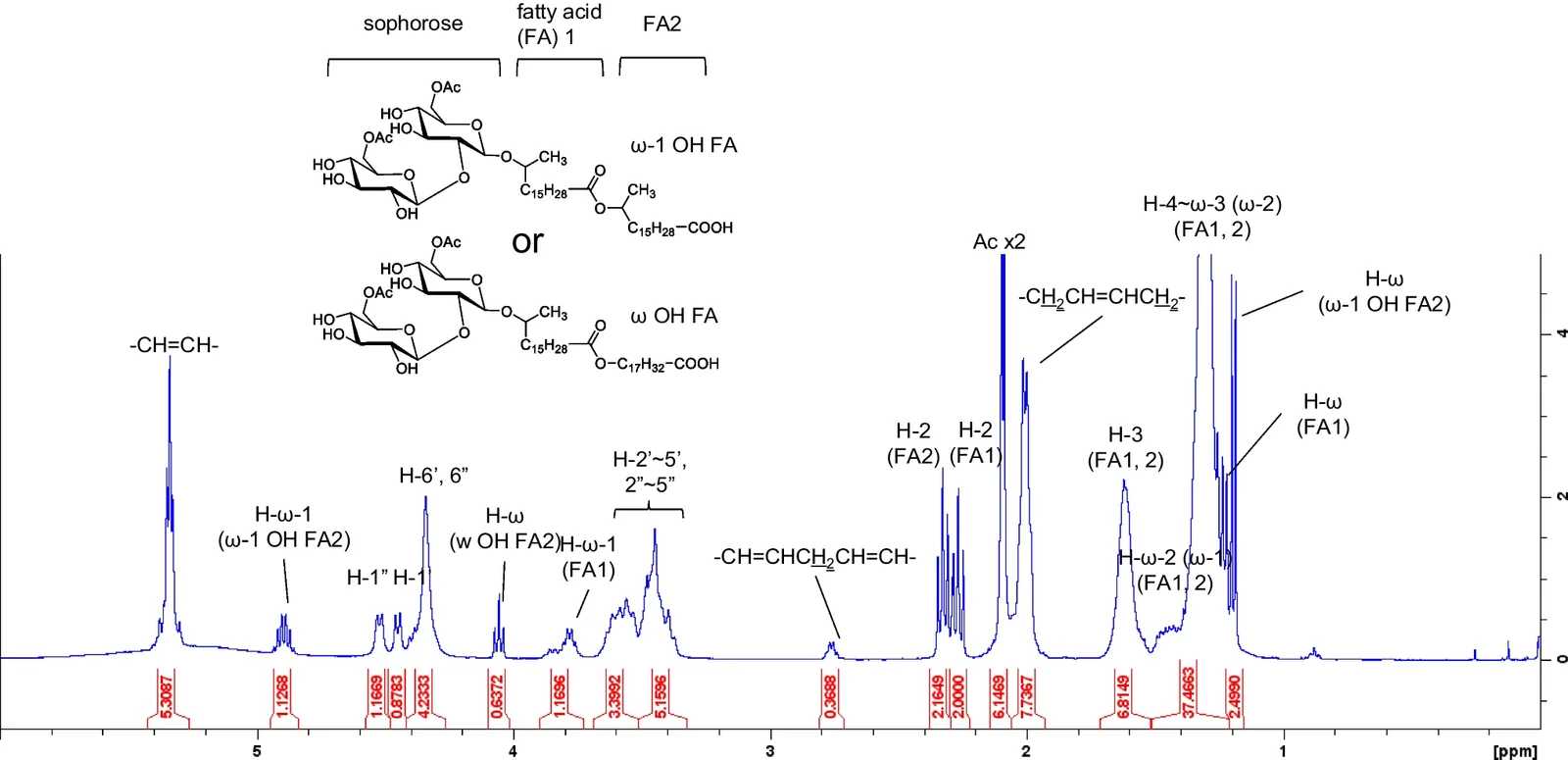
The 400-MHz proton nuclear magnetic resonance (^1^H-NMR) spectrum of compound Z, isolated from *S. bombicola*

Purified compound Z dissolved in chloroform, whereas the SL derivatives other than LSL did not. Detailed structural analysis of compound Z dissolved in deuterated chloroform was performed by 1D and 2D NMR analysis. The ^1^H NMR spectrum of compound Z (Fig. 4) was similar to that of the well-known ASL. Importantly, H-4'' of sophorose did not shift to a lower field, confirming that the hydroxyl group at the 4'' position was not esterified and neither lactone nor other esters formed. Similarly, it was confirmed that no other hydroxyl groups of sophorose (3', 4', 2'', 3'', and 4'' positions) had any ester bonds except for acetylation at 6' and 6'' positions. Focusing on the two triplet peaks from 2.2 to 2.4 ppm, which are not found in conventional ASLs, these peaks originate from the protons of the methylene group next to the carbonyl (i.e., carboxyl) groups (-CH_2_CO-). In particular, based on the difference in the positions of the two peaks, it is expected that one is a free carboxylic acid and the other is an ester.

**Fig. 5:**
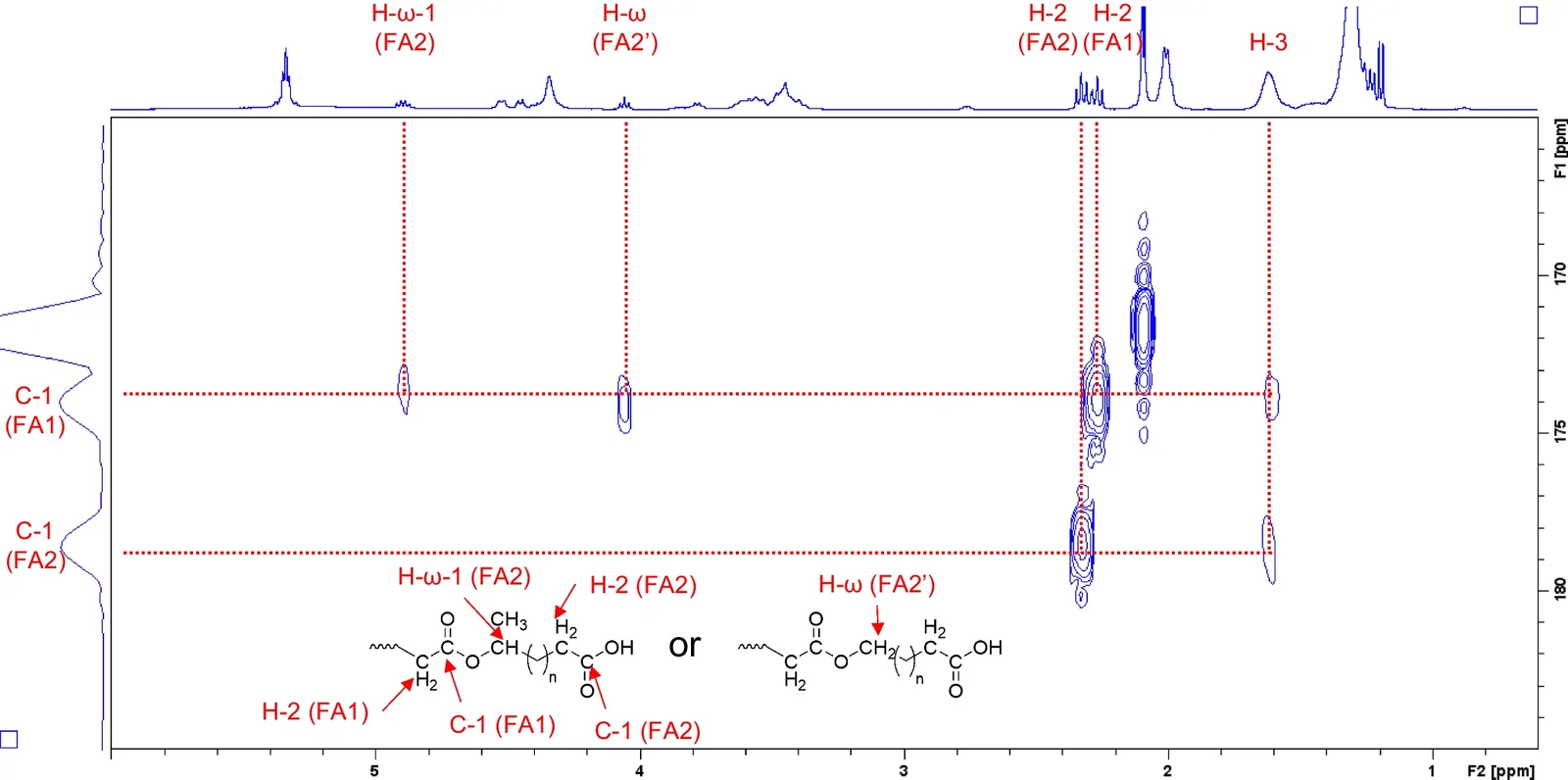
Partial heteronuclear multiple bond coherence (HMBC) spectrum of compound Z, isolated from *S. bombicola,* F1 axis: Carbon 13 NMR (^13^C-NMR) spectrum ranging from 165 to 185 ppm; F2 axis: ^1^H-NMR spectrum ranging from 0.6 to 6.0 ppm

It is important to note the other peaks at 4.06 and 4.89 ppm, which are not found in well-known ASLs. The multiplet peak at 4.89 ppm correlates with the doublet peak at 1.19 ppm (CH_3_CHOH-) and the broad peak at 1.5 ppm (-CH_2_-) in ^1^H-^1^H COSY (Fig. S5, Supplementary Information). This peak is associated with the proton at the ω-1 position, which has the same structure as the ω-1 position hydroxy fatty acid in the SL molecule. However, in conventional SLs, the hydroxyl group of the hydroxy fatty acid is connected to sophorose through an ether bond, whereas the proton at the ω-1 position appears at 3.78 ppm. The peak at 4.89 ppm was shifted toward the lower magnetic field; this implies that the hydroxy group at the ω-1 position is esterified. Based on these results, the peak at 4.89 ppm is derived from the proton of CH_3_CH(OCO-)-. Similarly, the triplet peak at 4.06 ppm is correlated with the broad peak at 1.61 ppm (-CH_2_-) in ^1^H-^1^H COSY (Fig. S11), derived from the proton at the ω position of ω hydroxy fatty acid; this hydroxyl group at the ω position is esterified (-CH_2_OCO-). Further, our HMBC analysis results (Fig. 5) confirmed that the carbon of the carboxyl group of the first fatty acid at 173.8 ppm (C = O of FA1) is correlated with the protons at 2.27 ppm (CH_2_C = O of FA1) and at 4.06 ppm (-CH_2_OCO- of the ω hydroxy FA2) or 4.89 ppm (CH_3_CH(OCO-)- of the ω-1 hydroxy FA2); these are the protons at the carbon to which the hydroxyl group of the second hydroxy fatty acid (FA2) is attached. In other words, our results showed that the carboxylic acid of ASL (FA1) forms an ester bond with the hydroxyl group of FA2. On the other hand, the carbon of the carboxyl group of the second fatty acid at 178.5 ppm (C = O of FA2) only correlates with the protons at 2.33 ppm (CH_2_C = O of FA2). Thus, the FA2 is a free carboxylic acid. Based on the above results, compound Z, which has [M-H]^–^ (m/z) of 985.6, the same carbohydrate structure as ASL, and two fatty acids (one of which is an ester and one of which is a free carboxylic acid) is estimated to have the structure shown in Fig. 3(c). The results of ^13^C NMR and HSQC (Fig. S12) analysis confirmed these findings (Table 3).

**Table 3:** NMR data for the compound Z (chloroform-*d*, 400 MHz)

	^13^C-NMR		^1^H-NMR	
	δ (ppm)		δ (ppm)	*J* (Hz)
Saccharides				
C-1'	100.6	H-1'	4.45 d	7.3
C-2'	81.3	H-2'	3.35–3.65 m	
C-3'	75.7 or 75.9	H-3'	3.35–3.65 m	
C-4'	69.8 or 70.4	H-4'	3.35–3.65 m	
C-5'	73.4 or 74.6	H-5'	3.35–3.65 m	
C-6'	63.5	H-6'	4.34 b	
C-1"	103.6	H-1"	4.52 d	7.3
C-2"	73.4 or 74.6	H-2"	3.35–3.65 m	
C-3"	75.7 or 75.9	H-3"	3.35–3.65 m	
C-4"	69.8 or 70.4	H-4"	3.35–3.65 m	
C-5"	73.4 or 74.6	H-5''	3.35–3.65 m	
C-6"	63.5	H-6"	4.34 b	
Acetyl groups				
–C = O (C-6',6")	171.6			
–CH_3_ (C-6',6")	20.9		2.09, 2.10 s	
Fatty acyl group 1 (FA1)				
–C = O	173.7			
–CO-CH_2_–	34.8		2.27 t	7.5
–CO-CH_2_CH_2_–	24.7		1.61 b	
–(CH_2_)_n_–	25.0–29.8		1.25–1.40 b	
–CH = CH-CH_2_–	27.1		2.00 b	
–CH = CH–	129.8		5.33 m	
–OCH(CH_3_)-CH_2_–	36.9		1.42 m	
–OCH(CH_3_)–	77.2		3.78 m	
–CH_3_	21.1		1.22 d	6.2
Fatty acyl group 2 (FA2)				
–C = O	178.5			
–CO-CH_2_–	34.0		2.33 t	7.5
–CO-CH_2_CH_2_–	24.7		1.61 b	
–(CH_2_)_n_–	25.0–29.8		1.25–1.40 b	
–CH = CH-CH_2_–	27.1		2.00 b	
–CH = CH–	129.8		5.33 m	
–OCH(CH_3_)-CH_2_–	36.9		1.45 m	
–OCH_2_OCO– (ω hydroxy acid)	64.5		4.06 t	6.7
–OCH(CH_3_)– (ω-1 hydroxy acid)	70.8		4.89 sextet	4.8
–CH_3_ (ω-1 hydroxy acid)	20.0		1.19 d	6.2

### Properties of compound Z

As mentioned above, compound Z has a completely unique chemical structure, in which two long-chain fatty acids are connected in series to a disaccharide; this feature is rare in common surfactants. We attempted to demonstrate the surface activity of this compound by measuring the surface tension of its aqueous solutions. When compound Z was dispersed in water, it became cloudy and formed an aggregate at lower concentrations (< 1 g/L), unlike ASL, which is highly soluble in water. Unexpectedly, we observed that even when compound Z was dispersed in water at a higher concentration (~ 20 g/L), the surface tension decreased only to 61.1 mN/m. Furthermore, in highly concentrated aqueous solutions (> 20 g/L), the insoluble part precipitated, and measurements could not be performed. From this result, compound Z has very low surface activity.

SLs are well known for excellent antimicrobial activity. We examined the antimicrobial activity of the newly found compound Z against gram-positive and gram-negative bacteria. Three bacteria (*B. subtilis*, *S. aureus*, and *E. coli*) were inoculated in liquid media containing SL derivatives, and the bacterial growth was observed. Compound Z did not exhibit antimicrobial activity against any bacteria in the concentration range tested (< 1,024 µg/mL), whereas LSL showed activity against gram-positive bacteria.

### Investigation of changes in component ratios in *S. bombicola* products

**Fig. 6:**
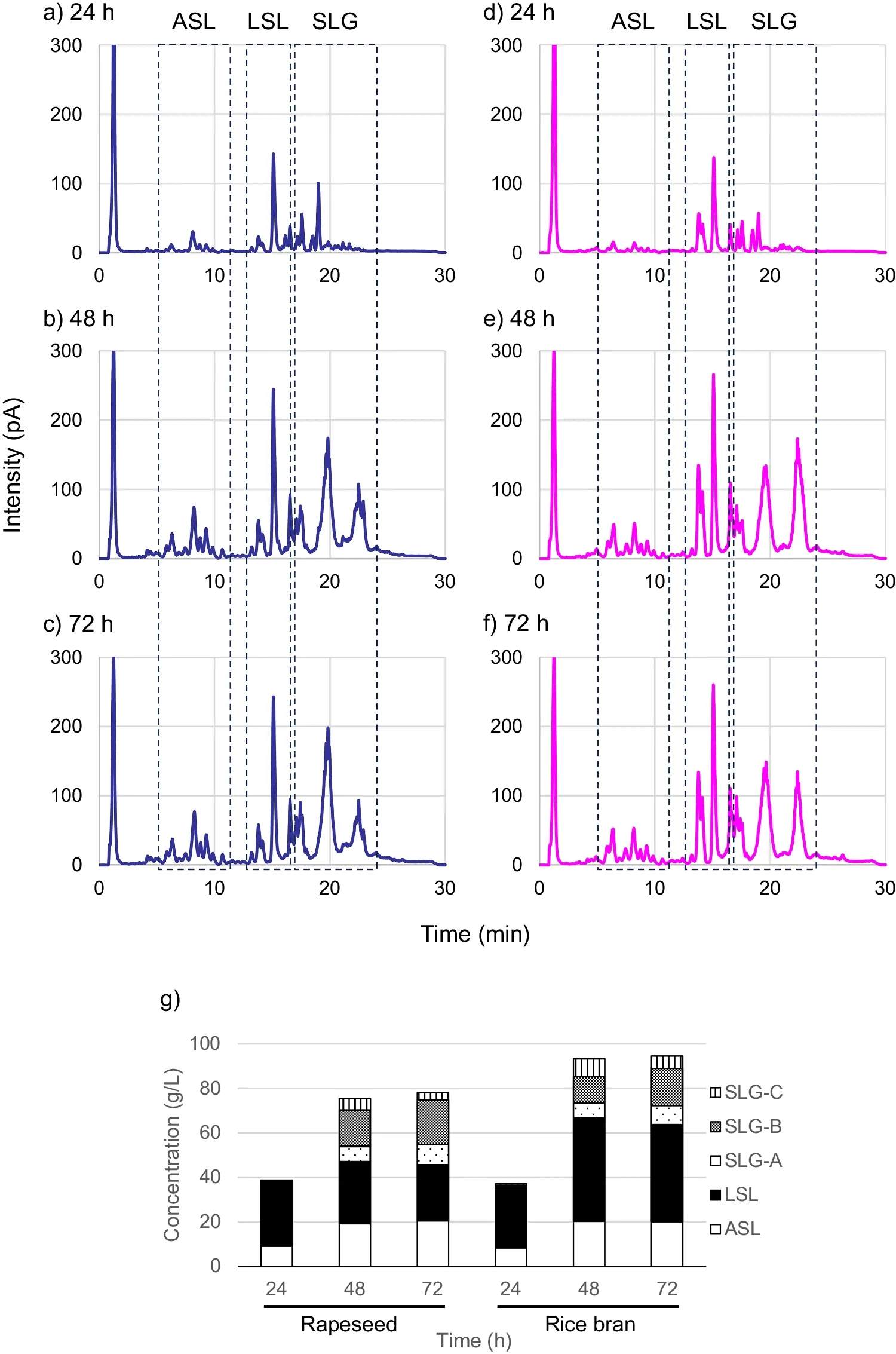
Component analysis of the culture medium of *S. bombicola* by CAD-HPLC using a C18 silica gel column and methanol/water eluent. Using rapeseed oil as a carbon resource at **a**) 24, **b**) 48, and **c**) 72 h, or using rice bran oil at **d**) 24, **e**) 48, and **f**) 72 h after the start of fermentation, and **g**) Quantification of the SL derivatives

As demonstrated, with the appropriate solvent, it is possible to analyze all components of SLs using HPLC and how the component composition changes over time in the cultured products of *S. bombicola*. Figure 6a–c show the changes in the components of *S. bombicola* products obtained from rapeseed oil after jar fermentation for 3 days. The culture solution was diluted directly with 70% methanol, filtered, and subjected to HPLC analysis. The quantification of SL derivatives and the culture profile are shown in Fig. 6g and Fig. S13 in the Supplementary Information. LSL (r.t. 11.5–16.8 min) was produced as the main component from the early stage of culture (~ 24 h). In contrast, the content ratio of the third component, the SLGs, (three peaks around r.t. 16.8–24 min) increased from the middle to late stages of culture (1.0 g/L at 24 h, 28.3 g/L at 48 h, and 32.4 g/L at 72 h), indicating that their accumulation in the culture solution was progressing. Furthermore, comparing the results after 48 and 72 h (Fig. 6b, c, and g), SLG-A–C tended to be converted to SLG-B (SL tri-glyceride: Fig. 1d, the peak at r.t. 19.3–20.5 min) (SLG-A:B:C = 7.0:16.2:5.1 [g/L] at 48 h, and 9.1:20.1:3.2 [g/L] at 72 h).

We changed the type of vegetable oil and used rice bran oil to track similar changes in the component ratio of *S. bombicola* products (Fig. 6d-f). The trend of the overall SL components was confirmed to be similar to that when rapeseed oil was used. That is, LSL was the main component from the early stage of culture throughout the entire period; SLGs increased from the middle to late stages of culture (1.9 g/L at 24 h, 26.5 g/L at 48 h, and 30.8 g/L at 72 h); SLG-A–C tended to be converted to SLG-B (SLG-A:B:C = 6.7:11.9:8.0 [g/L] at 48 h, and 8.6:16.7:5.6 [g/L] at 72 h). When rapeseed oil was used, the fatty acids constituting ASL and LSL were rich in C18:1 (r.t. 8–9 min and 15.2 min). However, when rice bran oil was used, the ratio of C18:2 and C16:0 (r.t. 6–7 min and 14 min) increased significantly.

## Discussion

There were two reasons why we worked on updating our SL component analysis method using HPLC. First, in the normal phase HPLC system (chloroform/methanol gradient solvent system) that we often used for our glycolipid analysis (Imura et al. 2014), the peaks of many hydrophilic components in *S. bombicola* products broadened out in the latter half of LC elution, and these peaks could not be separated sufficiently. Second, when attempting to perform analysis using a reversed-phase HPLC system, the commonly used acetonitrile/water gradient solvent system (Davila et al. 1993; Saerens et al. 2011; Ribeiro et al. 2012; Kotland et al. 2013; Kim et al. 2021) resulted in large amounts of undissolved SL components remaining in the analysis sample. Therefore, we went back to basics and confirmed the solubility of SL components in common solvents (Table 1). As expected, the SL derivatives had completely different solubilities in various solvents due to their different structures. Among the many types of solvents, we were able to confirm that methanol (lower alcohol) is a good solvent for all SL components, including the SL derivatives newly found in this study. Interestingly, it was confirmed that SL mixtures in *S. bombicola* products can be well dispersed in water. We speculate that this is probably a synergistic effect resulting from the mixture of the various SL components, including SLGs, found in this study.

By performing reversed-phase HPLC analysis using methanol as an eluent, each component can be separated and analyzed without leaking all SL components, and the constituent components can be identified in detail through linkage with the MS results. Thus, our approach enables qualitative and quantitative analysis of all SL components in SL products. Only two main SL derivatives, ASL and LSL, have hitherto been known; however, multiple derivatives, including SLGs that cannot be classified using conventional ASLs and LSLs, were identified in *S. bombicola* products in this study. Furthermore, due to differences in fermentation and collection conditions, *S. bombicola* products may contain vegetable oils, fatty acids, and sugars, etc. derived from feed materials. Therefore, if the recovered product was dissolved in methanol and filtered, it would be possible to analyze the components without leaking all of the SL components in the *S. bombicola* product.

To our knowledge, this is the first time that compound X (Fig. 3a), bola-form SL, has been identified in the product of wild-type *S. bombicola* and isolated from a naturally occurring SL product. Price et al. (2012) reported finding similar compounds in a natural SL mixture based on MS analysis; however, the compounds were not isolated, and their putative chemical structures differed from that of the bola-form SL in this study. The unique structure of bola-form SL, which has two disaccharides at both ends of a long alkyl chain, is expected to provide a specific self-assembling property for applications in nanotechnology and gene and drug delivery. The bola-form SL has been produced recently on a gram scale using the genetically engineered *S. bombicola* strain (Van Renterghem et al. 2018). It is our hope that bola-form SL is present in natural SL, which will provide valuable clues regarding its biosynthetic mechanism.

Additionally, SL mono-glyceride (compound Y: Fig. 3b) was not found in our previous report on SL di- and tri-glycerides (Kobayashi et al. 2023). Its existence was predicted based on the findings of Nuñez et al. (2001), who found that it was a trace component in their MS analysis of SLs. We were able to isolate and identify compound Y from a *S. bombicola* product for the first time in the present study. This compound is a highly soluble SL derivative in water and many polar organic solvents. Moreover, compound Y is less sensitive to pH as it is a nonionic surfactant, unlike ASL. Currently, this mono-glyceride is still a trace component. We expect that production conditions will be investigated to increase the content of this compound in SL products and that analysis of its physical properties, both individually and as a mixture of SL derivatives, will be performed in the future.

Compound Z (Fig. 3c) is a completely new SL derivative that has not hitherto been reported. Specifically, this is the first report of compound Z in a *S. bombicola* product, which our group isolated, identified, and characterized. This compound may be classified as an ASL from a structural point of view; however, it is nearly insoluble in water, whereas ASL is highly water-soluble. In addition, compound Z shows little surface tension-lowering activity in water (~ 61.1 mN/m). It is presumed that the two linked long-chain fatty acids are highly hydrophobic, and that even if there is a disaccharide at the end, it has poor amphiphilic properties. In our previous study on the isolation and characterization of SLGs (Kobayashi et al. 2023), only SLG-C (Fig. 1e) like fats and oils did not show surface activity, unlike SLG-A (Fig. 1c) and SLG-B (Fig. 1d). Compound Z may also exhibit more lipophilic properties among SL components in *S. bombicola* products; however, it did not show antibacterial activity. In a previous study, we demonstrated the excellent antimicrobial activity of SLs due to LSL, and that the activity levels of ASL and SLGs are not high; the results of the current study are in good agreement with our earlier findings (Kobayashi et al. 2023).

The present analysis method enables direct component analysis of culture media of *S. bombicola* by simply diluting with methanol and filtering (Fig. 6). We were able to closely track changes in composition over the course of fermentation. In particular, we were able to confirm production of the well-known LSL and ASL in the early stage, the gradual accumulation of SLGs in the middle stage, and the conversion of SLGs to SLG-B in the final stage. According to the culture profile (Fig. S13a) and the quantitative results of SL production (Fig. 6g), the carbon sources (glucose and oil) were nearly consumed completely after 48 h; cell growth (OD_600_) and SL production reached a plateau after this point. Therefore, it is presumed that conversion to SLG-B is progresses through extracellular reactions, such as transesterification in the late stage of culture. In addition, we were able to accurately track the composition of fatty acyl groups of SL components. While the main fatty acid component of SL from rapeseed oil is C18:1, more C18:2 and C16:0 fatty acids were detected in the SLs from rice bran oil. The main fatty acid component of rapeseed oil is oleic acid (C18:1; ca. 63%), whereas rice bran oil has oleic acid (C18:1; ca 42%) and the second highest content ratio of linoleic acid (C18:2; ca. 33%) and palmitic acid (C16:0; ca. 20%) (Orsavova et al. 2015; Rudzińska et al. 2016). Because the constituent SL fatty acids are strongly influenced by the vegetable oil used as a raw material, the present results reflect this.

As described above, our analysis method provides a simple and thorough means of analyzing the constituent components of SLs and how they change during fermentation. The insights into SLs gained through the use of our approach will serve to advance SL research and development efforts in the future.

## Supplementary Information

Below is the link to the electronic supplementary material.

Supplementary file1 (PDF 766 KB)

## Data Availability

All data generated or analyzed during this study are included in this published article and its supplementary information file.
