# Supplementary material for: Updated component analysis method for naturally occurring sophorolipids from Starmerella bombicola
Source: Appl Microbiol Biotechnol. 2024 Apr 12;108:296. doi: 10.1007/s00253-024-13138-x (PMC11009742; doi:10.1007/s00253-024-13138-x)
Supplement: Supplementary file 1 [file 253_2024_13138_MOESM1_ESM.pdf]

**Supplementary Information (SI)**

**Applied Microbiology and Biotechnology**

# Updated component analysis method for naturally occurring sophorolipids from *Starmerella bombicola*

Yosuke Kobayashi[1], Qiushi Li[1], Kazunori Ushimaru[2], Makoto Hirota[1], Tomotake Morita[2], Tokuma Fukuoka[2]*

[1] Allied Carbon Solutions Co., Ltd., 847-1 Ozuwa, Numazu, Shizuoka 410-0873, Japan

[2] Research Institute for Sustainable Chemistry, National Institute of Advanced Industrial Science and Technology (AIST), Tsukuba Central 5-2, 1-1-1, Higashi, Tsukuba, Ibaraki 305-8565, Japan

*Corresponding author

(TEL: +81-50-3521-1265; E-mail: t-fukuoka@aist.go.jp)

ORCID: 0000-0003-1814-7046

**Table of contents**

**Supplementary Tables**

**Table S1** NMR data for the compound X (methanol-*d4*, 400 MHz)

**Table S2** NMR data for the compound Y (methanol-*d4*, 400 MHz)

**Supplementary Figures**

**Fig. S1** The numbered chemical structures of compound X, Y, and Z

**Fig. S2** LC-MS analysis of compound X, isolated from *S. bombicola*

Total ion chromatogram (TIC) and mass spectrum

**Fig. S3** The 400 MHz $^{1}$H-NMR spectrum of compound X, isolated from *S. bombicola*

**Fig. S4** Partial $^{1}$H-$^{1}$H COSY spectrum of compound X, isolated from *S. bombicola*

F1 axis: $^{1}$H-NMR spectrum ranging from 3.0 to 6.0 ppm; F2 axis: $^{1}$H-NMR spectrum ranging from 3.0 to 6.0 ppm

**Fig. S5** HSQC spectrum of compound X, isolated from *S. bombicola*

F1 axis: $^{13}$C-NMR spectrum ranging from 10 to 140 ppm; F2 axis: $^{1}$H-NMR spectrum ranging from 0.8 to 6.0 ppm

**Fig. S6** LC-MS analysis of compound Y, isolated from *S. bombicola*.

**Fig. S7** The 400 MHz $^{1}$H-NMR spectrum of compound Y, isolated from *S. bombicola*

**Fig. S8** Partial $^{1}$H-$^{1}$H COSY spectrum of compound Y, isolated from *S. bombicola*

F1 axis: $^{1}$H-NMR spectrum ranging from 3.0 to 4.8 ppm; F2 axis: $^{1}$H-NMR spectrum ranging from 3.0 to 4.8 ppm

**Fig. S9** HSQC spectrum of compound Y, isolated from *S. bombicola*

F1 axis: $^{13}$C-NMR spectrum ranging from 10 to 140 ppm; F2 axis: $^{1}$H-NMR spectrum ranging from 0.8 to 5.6 ppm

**Fig. S10** LC-MS analysis of compound Z, isolated from *S. bombicola*.

**Fig. S11** Partial $^{1}$H-$^{1}$H COSY spectrum of compound Z, isolated from *S. bombicola*

F1 axis: $^{1}$H-NMR spectrum ranging from 1.0 to 1.8 ppm; F2 axis: $^{1}$H-NMR spectrum ranging from 3.0 to 5.5 ppm

**Fig. S12** HSQC spectrum of compound Z, isolated from *S. bombicola*

F1 axis: $^{13}$C-NMR spectrum ranging from 10 to 140 ppm; F2 axis: $^{1}$H-NMR spectrum ranging from 0.8 to 5.6 ppm

**Fig. S13** Culture profiles of jar fermentation of *S. bombicola*

(a) Rapeseed oil, (b) rice bran oil; circle: glucose; triangle: oil; square: $OD_{600}$

**Table S1** NMR data for the compound X (methanol-*d4*, 400 MHz)

| | $^{13}$C-NMR δ (ppm) | | $^{1}$H-NMR δ (ppm) | *J* (Hz) |
|---|---|---|---|---|
| **Saccharides (G1~4)** | | | | |
| C-1 | 92.6 (G3),<br>101.2 (G1),<br>104.2 (G2,4) | H-1 | 4.48 d (G1),<br>4.59 d (G2,4),<br>5.63 d (G3) | 7.7 |
| C-2 | 74.6 (G2,4),<br>81.1 (G3),<br>82.4 (G1), | H-2 | 3.58 t (G3),<br>3.20-3.65 m | 9.0 |
| C-3 | 76.1–76.8 | H-3 | 3.20–3.65 m | |
| C-4 | 69.3–70.0 | H-4 | 3.20–3.65 m | |
| C-5 | 73.5–74.2 | H-5 | 3.20–3.65 m | |
| C-6 | 60.8–61.3 | H-6 | 3.70, 3.85 m | |
| C-6 (acetylated) | 63.4–63.6 | H-6 (acetylated) | 4.22, 4.39 m | |
| **Acetyl groups** | | | | |
| –C=O (C-6) | 171.6 | | | |
| $–CH_3$ (C-6) | 19.4, 19.6 | | 2.07, 2.09 s | |
| **Acyl group** | | | | |
| –C=O | 173.7 | | | |
| $–CO-\underline{CH_2}–$ | 33.5 | | 2.44 m | |
| $–CO-CH_2\underline{CH_2}–$ | 24.2 | | 1.64 b | |
| $–(CH_2)_n–$ | 24.8–29.6 | | 1.3–1.4 b | |
| $–CH=CH-\underline{CH_2}–$ | 26.8 | | 2.0–2.1 b | |
| –CH=CH– | 129.5 | | 5.37 m | |
| $–OCH(CH_3)-\underline{CH_2}–$ | 36.5 | | 1.64 b | |
| $–O\underline{CH}(CH_3)–$ | 77.3 | | 3.74 m | |
| $–CH_3$ | 20.5 | | 1.22 d | 6.2 |

**Table S2** NMR data for the compound Y (methanol-*d4*, 400 MHz)

| | $^{13}$C-NMR δ (ppm) | | $^{1}$H-NMR δ (ppm) | *J* (Hz) |
|---|---|---|---|---|
| **Saccharides** | | | | |
| C-1' | 101.2 | H-1' | 4.47 d | 7.7 |
| C-2' | 82.4 | H-2' | 3.2–3.5 m | |
| C-3' | 76.1 or 76.4 | H-3' | 3.2–3.5 m | |
| C-4' | 70.0 or 70.1 | H-4' | 3.2–3.5 m | |
| C-5' | 73.5 or 74.2 | H-5' | 3.2–3.5 m | |
| C-6' | 63.4 or 63.5 | H-6'a | 4.21 m | |
| | | H-6'b | 4.37 m | |
| C-1'' | 104.3 | H-1'' | 4.57 d | 7.8 |
| C-2'' | 74.7 | H-2'' | 3.2–3.5 m | |
| C-3'' | 76.1 or 76.4 | H-3'' | 3.2–3.5 m | |
| C-4'' | 70.0 or 70.1 | H-4'' | 3.2–3.5 m | |
| C-5'' | 73.5 or 74.2 | H-5'' | 3.2–3.5 m | |
| C-6'' | 63.4 or 63.5 | H-6''a | 4.21 m | |
| | | H-6''b | 4.37 m | |
| **Acetyl groups** | | | | |
| –C=O (C-6',6'') | 171.3 | | | |
| –$CH_3$ (C-6',6'') | 19.4, 19.5 | | 2.06, 2.08 s | |
| **Glycerol groups** | | | | |
| –$CH_2$O– | 65.1 | | 4.08, 4.15 m | |
| –CHO– | 69.7 | | 3.85 m | |
| –$CH_2$OH | 62.7 | | 3.57 m | |
| **Acyl group** | | | | |
| –C=O | 174.2 | | | |
| –CO-$\underline{CH_2}$– | 33.6 | | 2.37 t | 7.4 |
| –CO-$CH_2\underline{CH_2}$– | 24.6 | | 1.62 b | |
| –$(CH_2)_n$– | 24.7–29.6 | | 1.2–1.4 b | |
| –CH=CH-$\underline{CH_2}$– | 26.8 | | 2.0–2.1 b | |
| –CH=CH– | 129.5 | | 5.36 m | |
| –OCH($CH_3$)-$\underline{CH_2}$– | 36.5 | | 1.62 b | |
| –O$\underline{CH}$($CH_3$)– | 77.2 | | 3.76 m | |
| –$CH_3$ | 20.5 | | 1.21 d | 6.2 |

**Fig. S1**

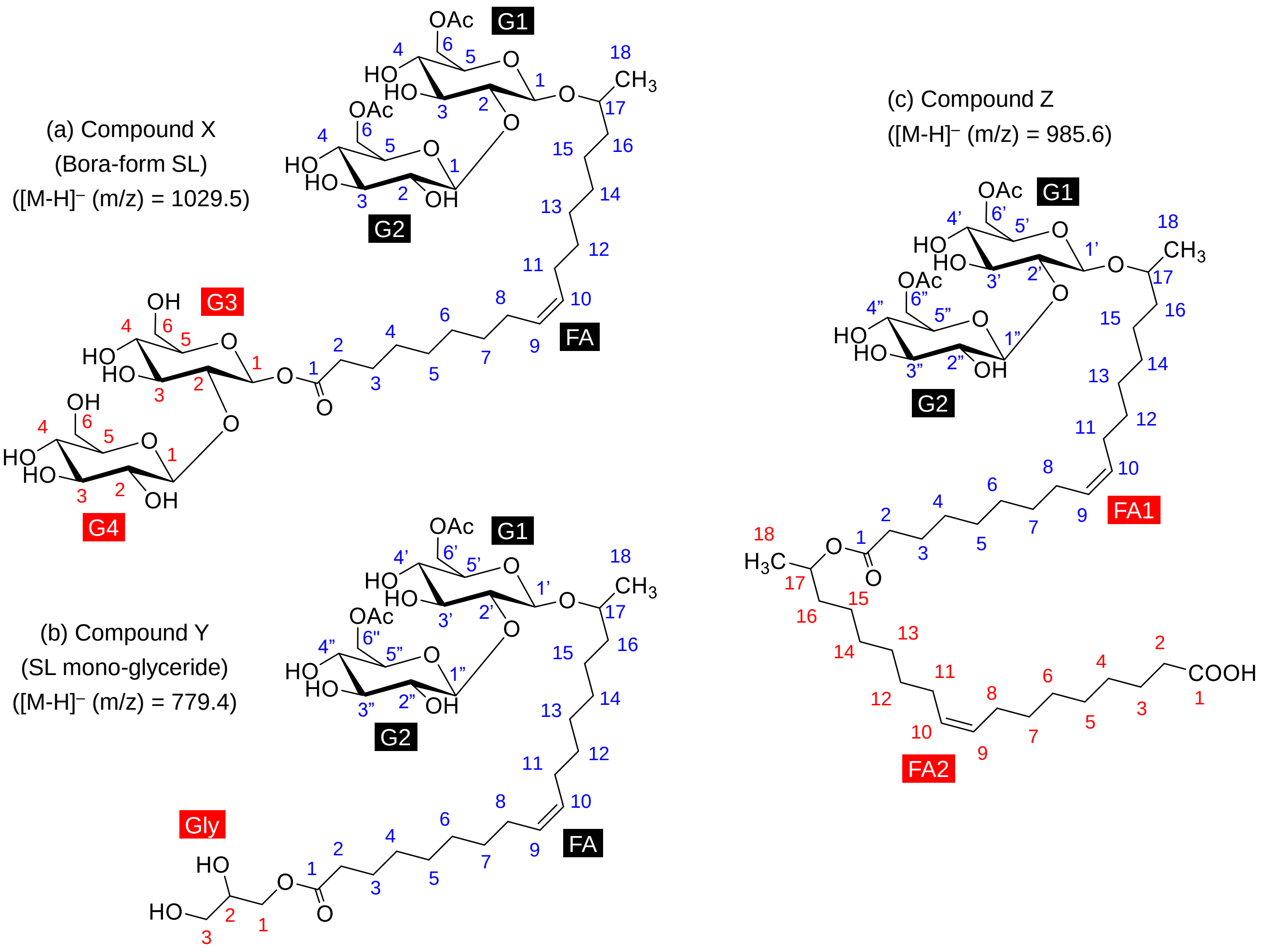
(a) Compound X
(Bora-form SL)
([M-H]⁻ (m/z) = 1029.5)
G1
G2
G3
G4
FA
(b) Compound Y
(SL mono-glyceride)
([M-H]⁻ (m/z) = 779.4)
G1
G2
Gly
FA
(c) Compound Z
([M-H]⁻ (m/z) = 985.6)
G1
G2
FA1
FA2

**Fig. S2**

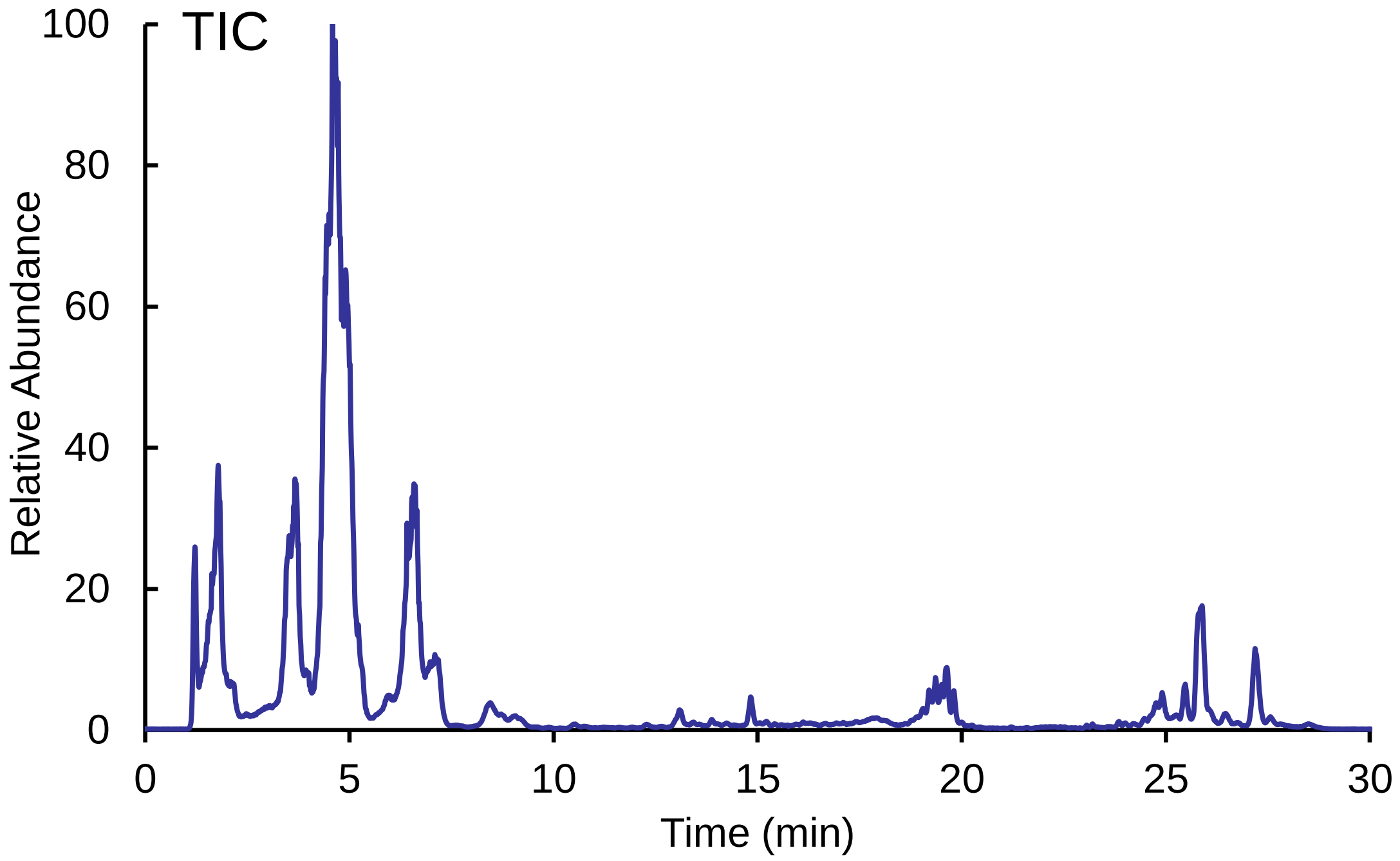
TIC
Relative Abundance
100
80
60
40
20
0
0
5
10
15
20
25
30
Time (min)

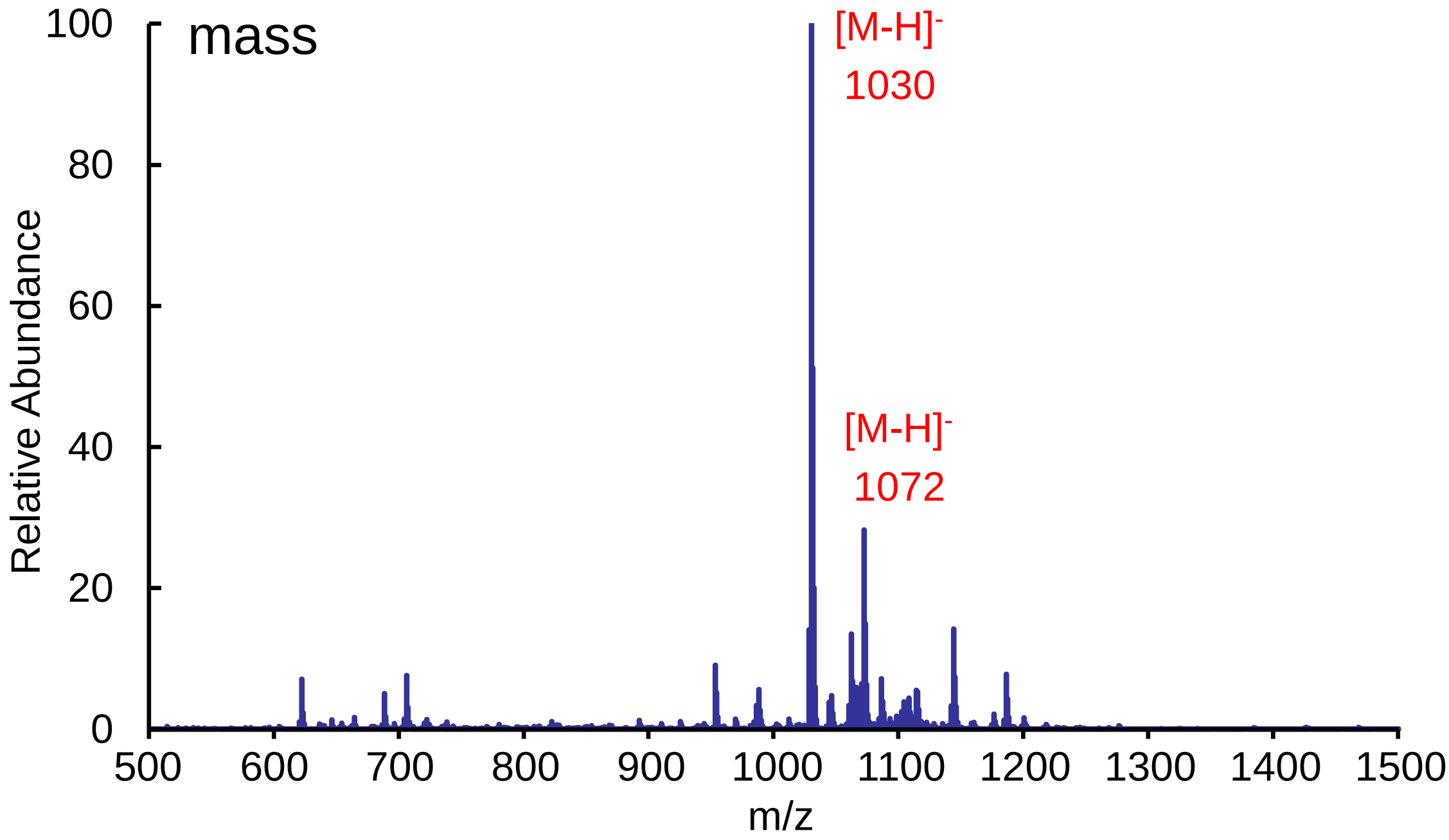
mass
Relative Abundance
[M-H]-
1030
[M-H]-
1072
100
80
60
40
20
0
500
600
700
800
900
1000
1100
1200
1300
1400
1500
m/z

**Fig. S3**

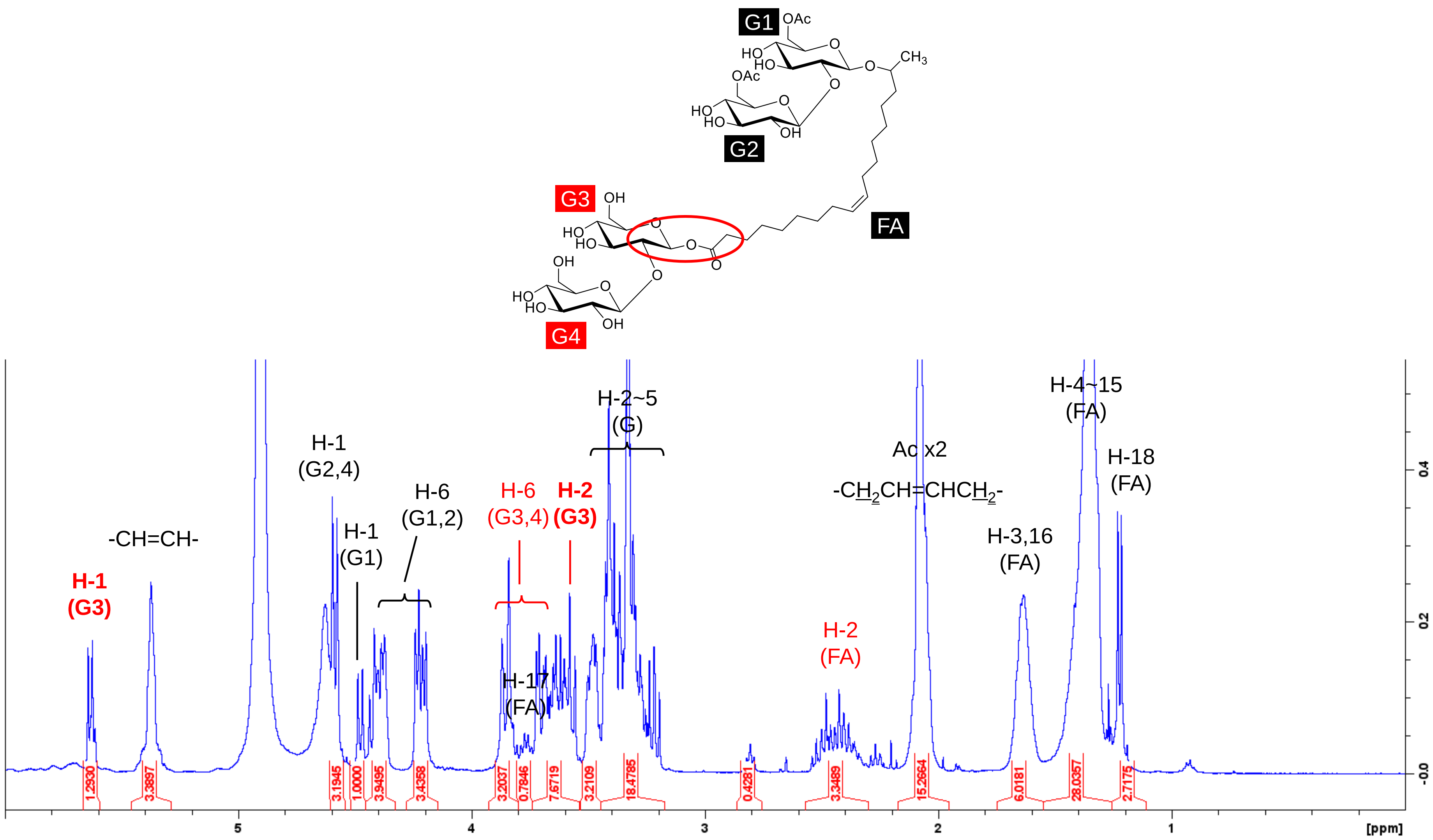
G1
OAc
HO
HO
O
O
CH3
OAc
O
O
HO
HO
OH
G2
G3
OH
HO
HO
O
O
O
O
FA
OH
HO
HO
O
OH
G4
H-1
(G3)
-CH=CH-
H-1
(G2,4)
H-1
(G1)
H-6
(G1,2)
H-6
(G3,4)
H-2
(G3)
H-17
(FA)
H-2~5
(G)
H-2
(FA)
Ac x2
-CH2CH=CHCH2-
H-3,16
(FA)
H-4~15
(FA)
H-18
(FA)
1.2930
3.3897
3.1945
1.0000
3.9495
3.4358
3.2037
0.7846
7.6719
3.2109
18.4785
0.4281
3.3489
15.2664
6.0181
28.0357
2.7175
5
4
3
2
1
[ppm]
0.4
0.2
-0.0

**Fig. S4**

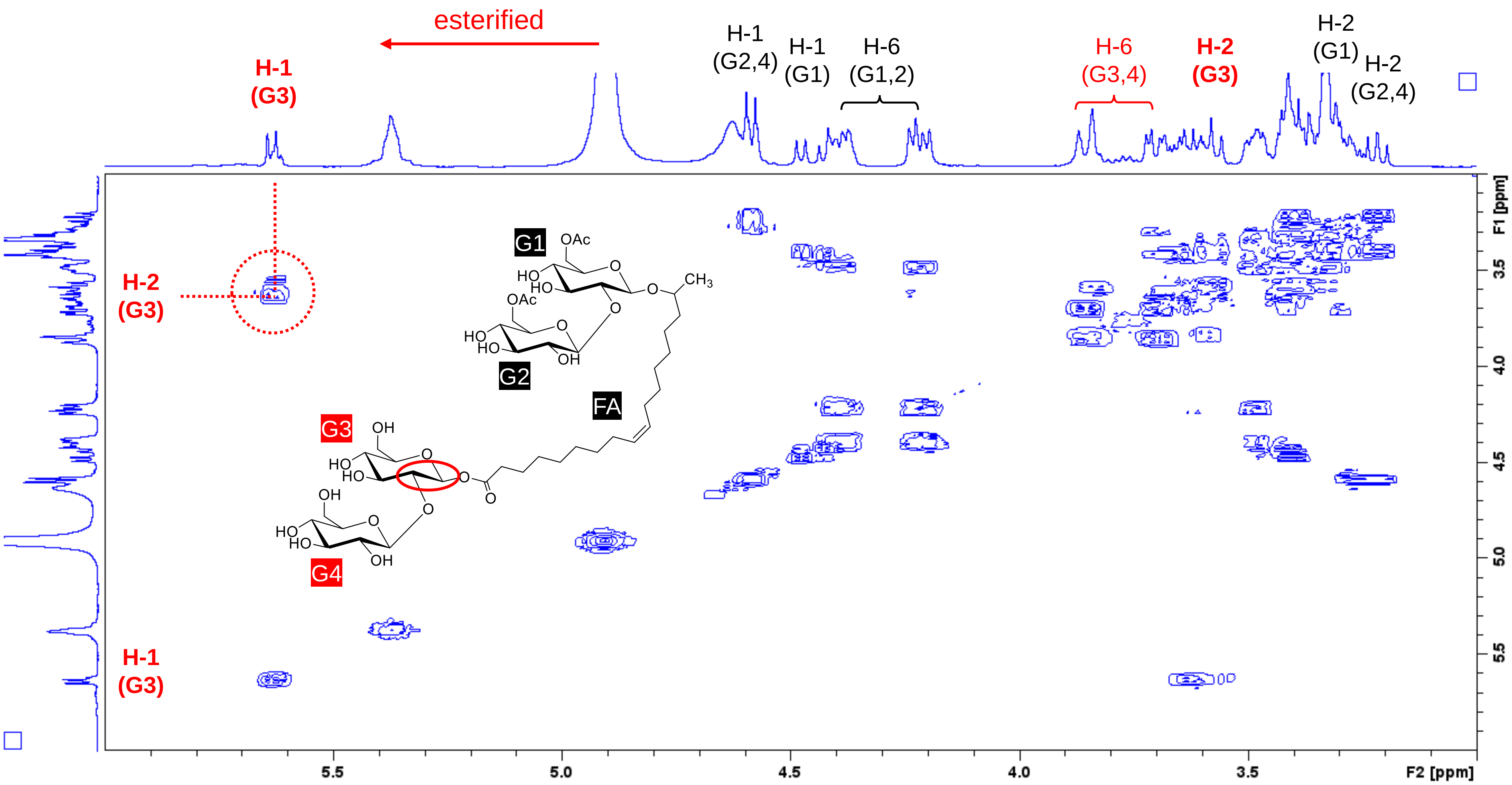
esterified
H-1
(G3)
H-1
(G2,4)
H-1
(G1)
H-6
(G1,2)
H-6
(G3,4)
H-2
(G3)
H-2
(G1)
H-2
(G2,4)
H-2
(G3)
H-1
(G3)
G1
OAc
HO
HO
O
CH3
OAc
HO
HO
OH
G2
FA
G3
OH
HO
HO
O
OH
HO
HO
OH
G4
F1 [ppm]
3.5
4.0
4.5
5.0
5.5
5.5
5.0
4.5
4.0
3.5
F2 [ppm]

**Fig. S5**

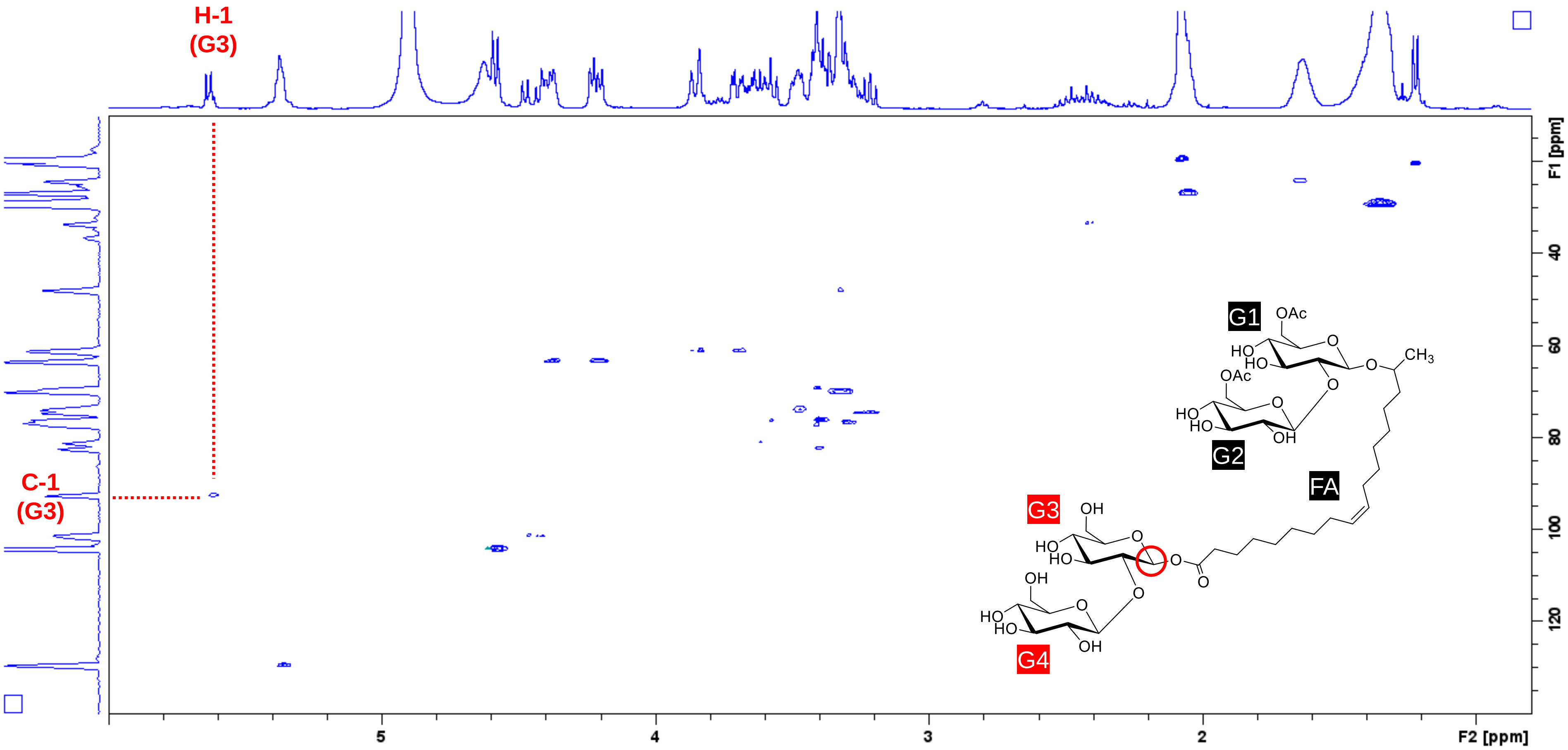
H-1
(G3)
C-1
(G3)
F1 [ppm]
40
60
80
100
120
5
4
3
2
F2 [ppm]
G1
OAc
HO
HO
O
O
CH$_3$
OAc
HO
HO
O
O
OH
G2
FA
G3
OH
HO
HO
O
O
O
O
OH
HO
HO
O
OH
G4

**Fig. S6**

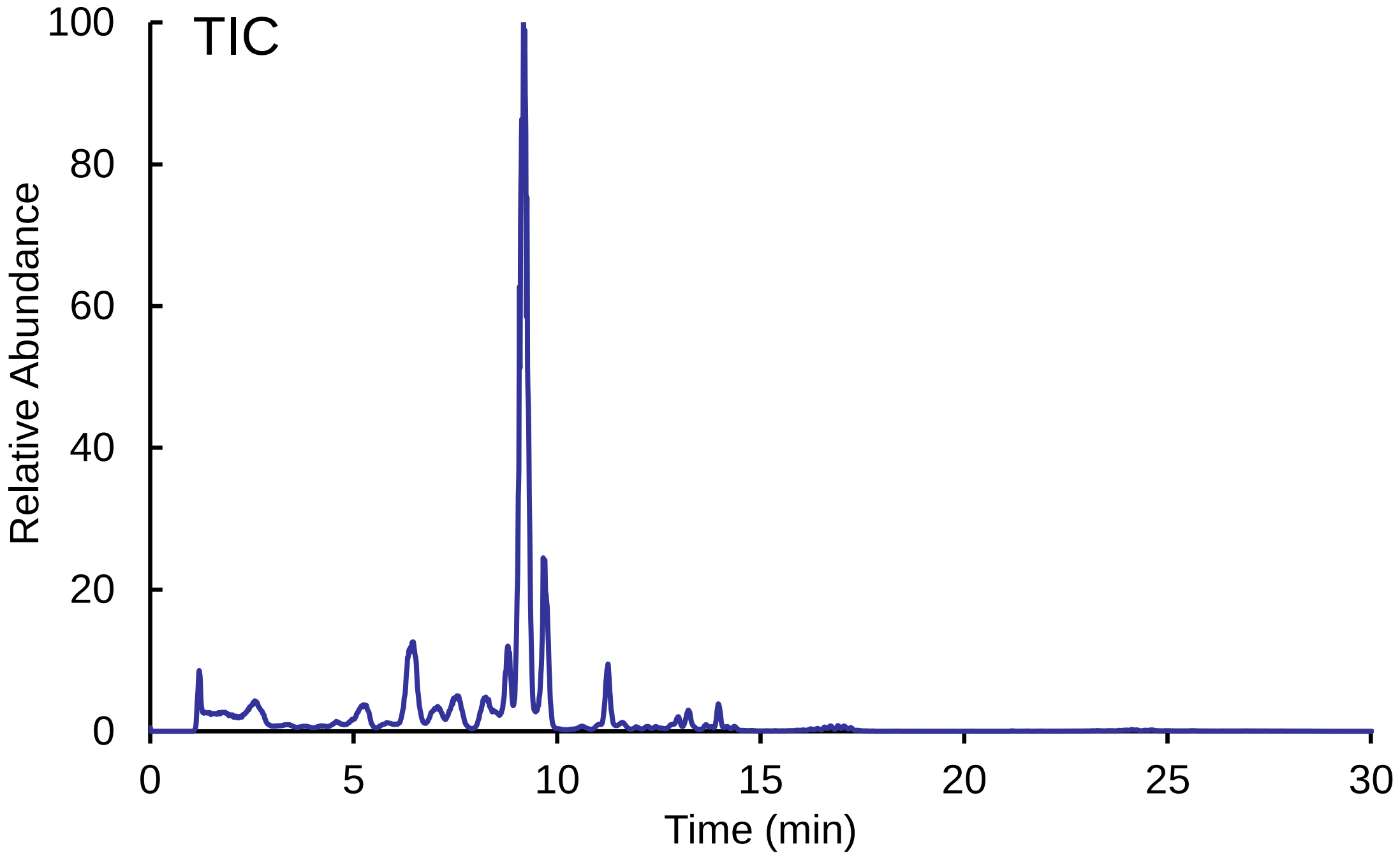

TIC
Relative Abundance
100
80
60
40
20
0
0
5
10
15
20
25
30
Time (min)


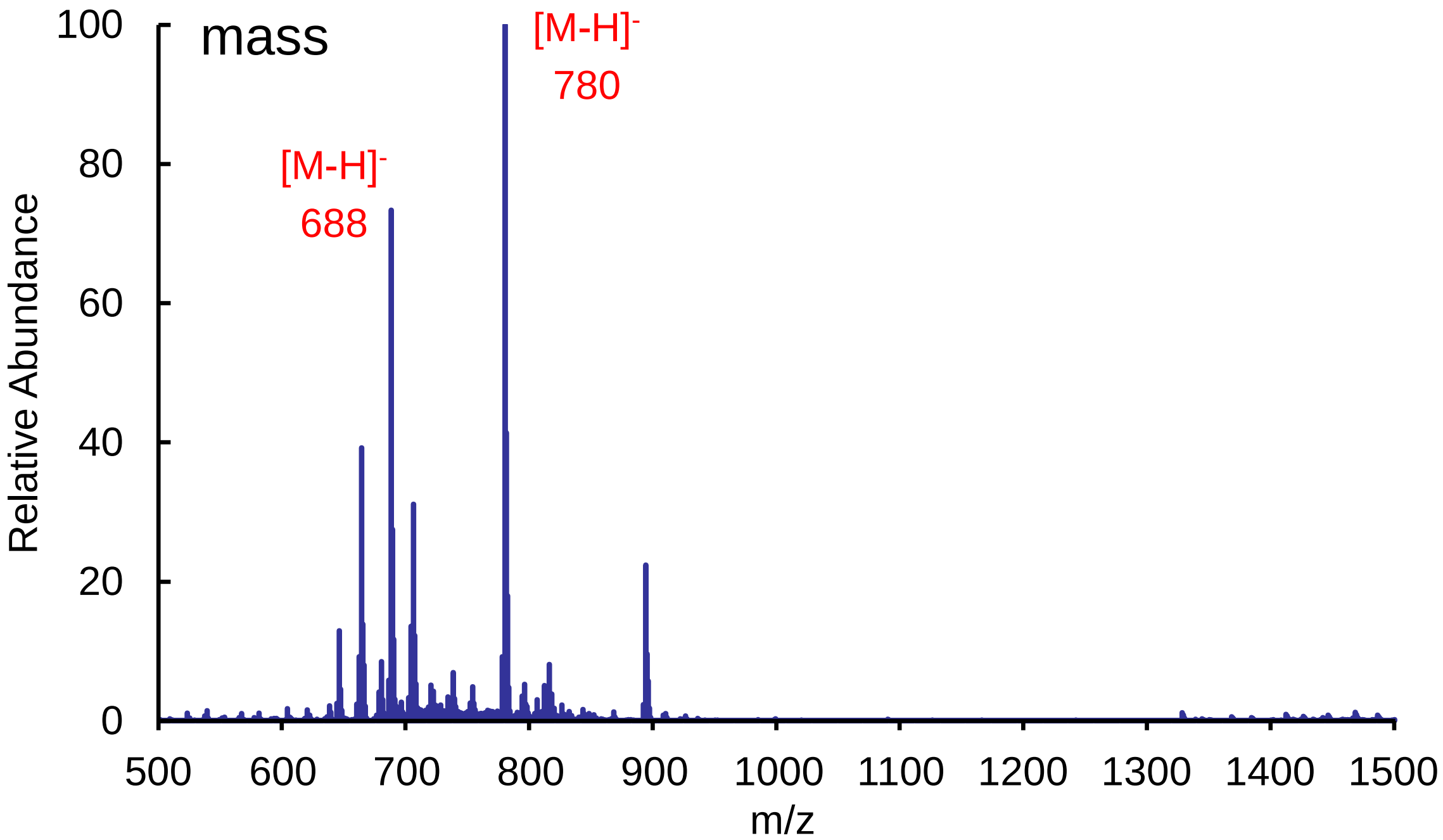

mass
[M-H]-
780
[M-H]-
688
Relative Abundance
100
80
60
40
20
0
500
600
700
800
900
1000
1100
1200
1300
1400
1500
m/z

**Fig. S7**

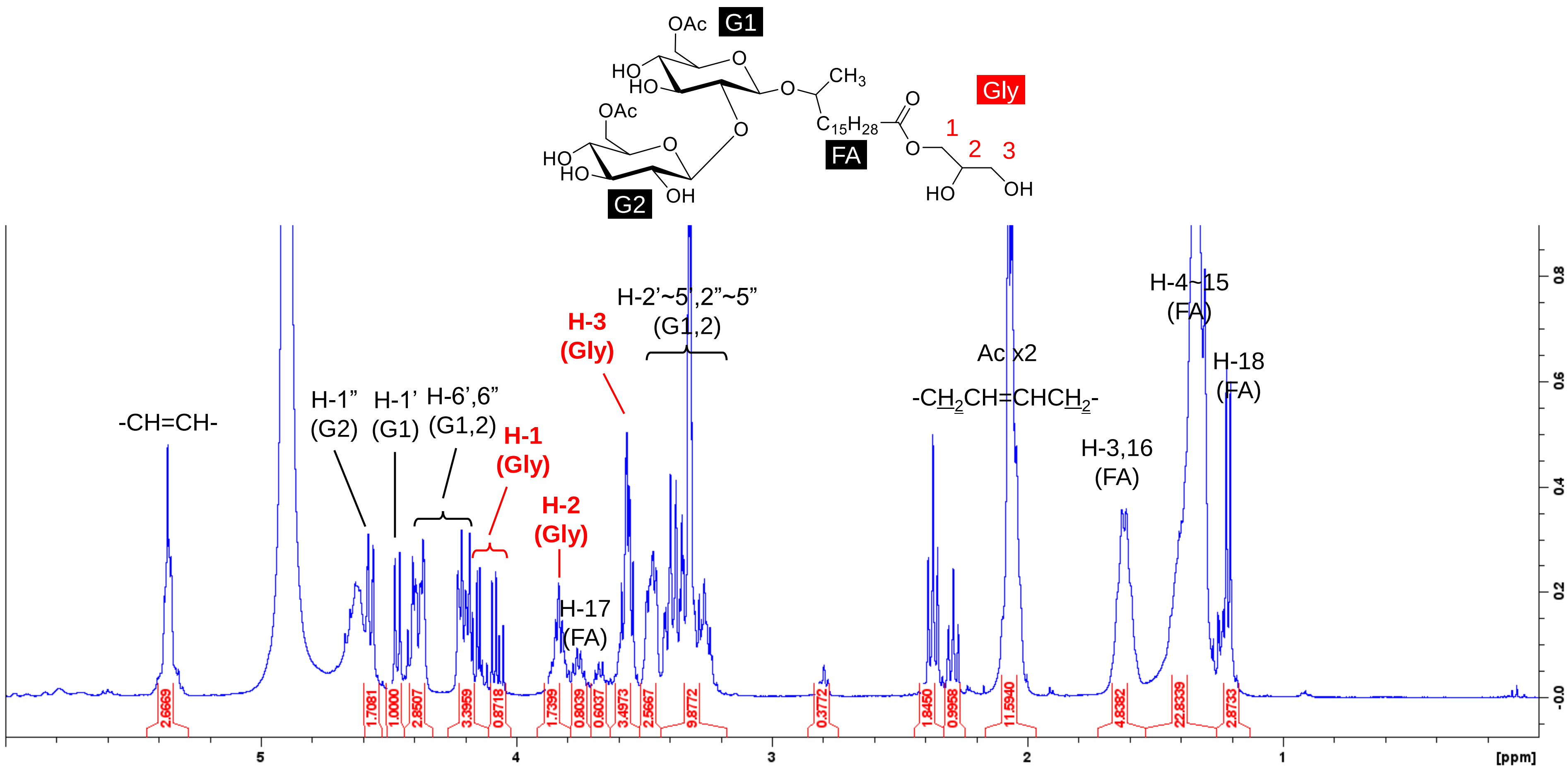

OAc
G1
HO
HO
O
O
CH3
OAc
O
O
Gly
C15H28
1
2
3
HO
HO
O
FA
O
G2
OH
HO
OH
H-2'~5',2"~5"
(G1,2)
H-3
(Gly)
H-4~15
(FA)
Ac x2
H-18
(FA)
-CH2CH=CHCH2-
-CH=CH-
H-1"
(G2)
H-1'
(G1)
H-6',6"
(G1,2)
H-1
(Gly)
H-3,16
(FA)
H-2
(Gly)
H-17
(FA)
[ppm]

**Fig. S8**

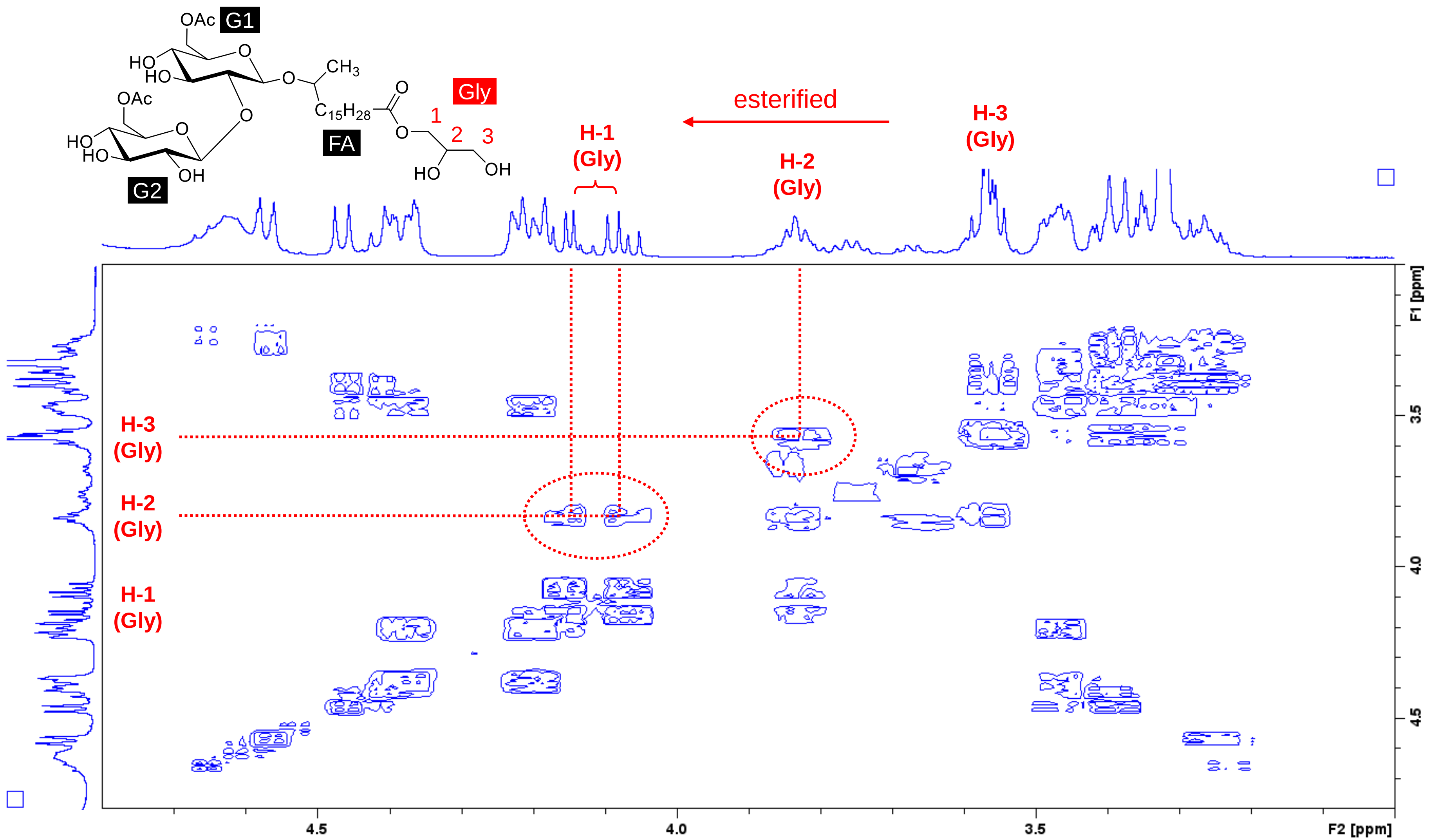

OAc
G1
HO
HO
O
O
$CH_3$
OAc
O
HO
HO
O
OH
G2
$C_{15}H_{28}$
O
FA
O
1
2
3
HO
OH
Gly
esterified
H-1 (Gly)
H-2 (Gly)
H-3 (Gly)
H-3 (Gly)
H-2 (Gly)
H-1 (Gly)
F1 [ppm]
3.5
4.0
4.5
4.5
4.0
3.5
F2 [ppm]

**Fig. S9**

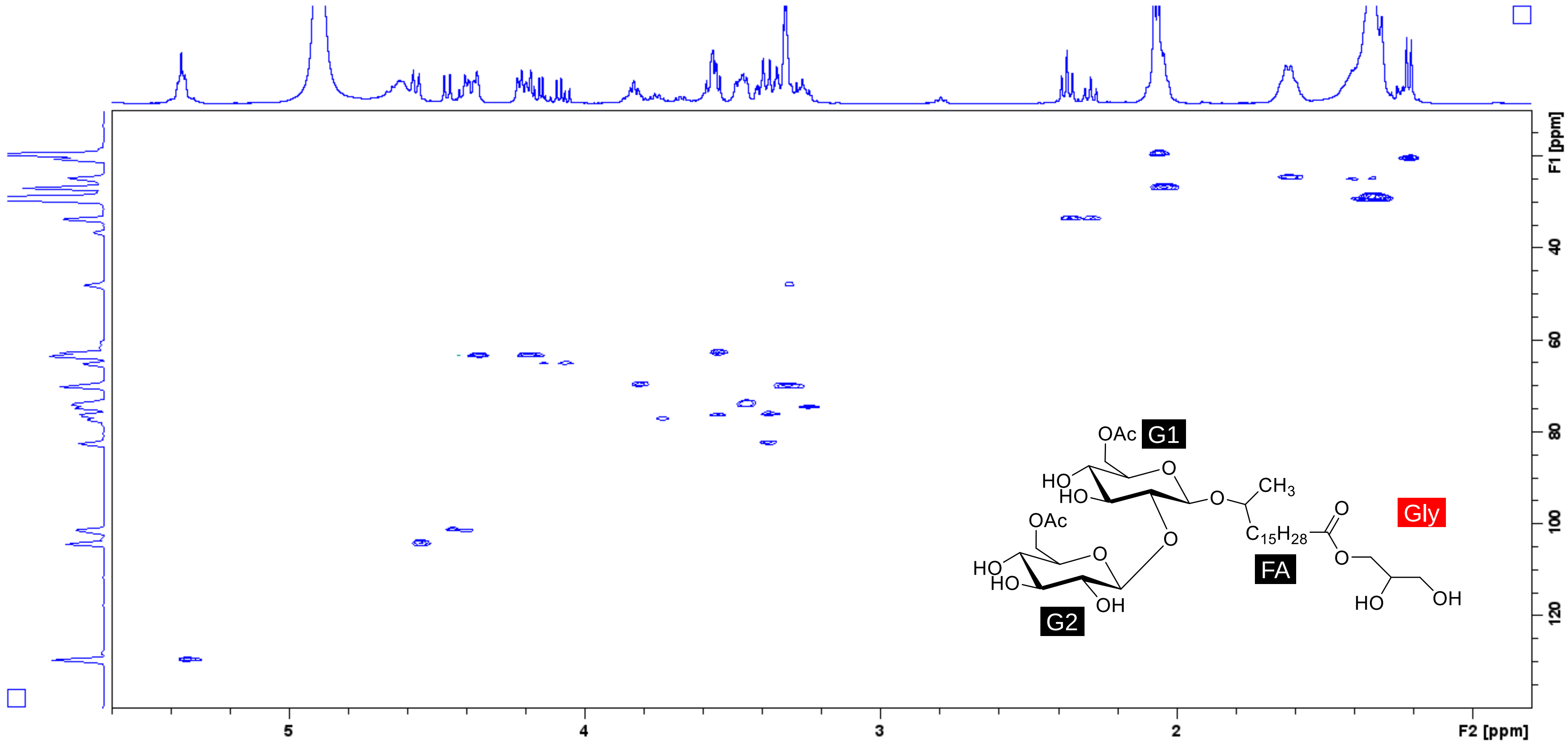

F1 [ppm]
40
60
80
100
120
5
4
3
2
F2 [ppm]
OAc
G1
HO
HO
O
O
CH3
Gly
OAc
O
O
C15H28
HO
HO
OH
FA
O
HO
OH
G2

**Fig. S10**

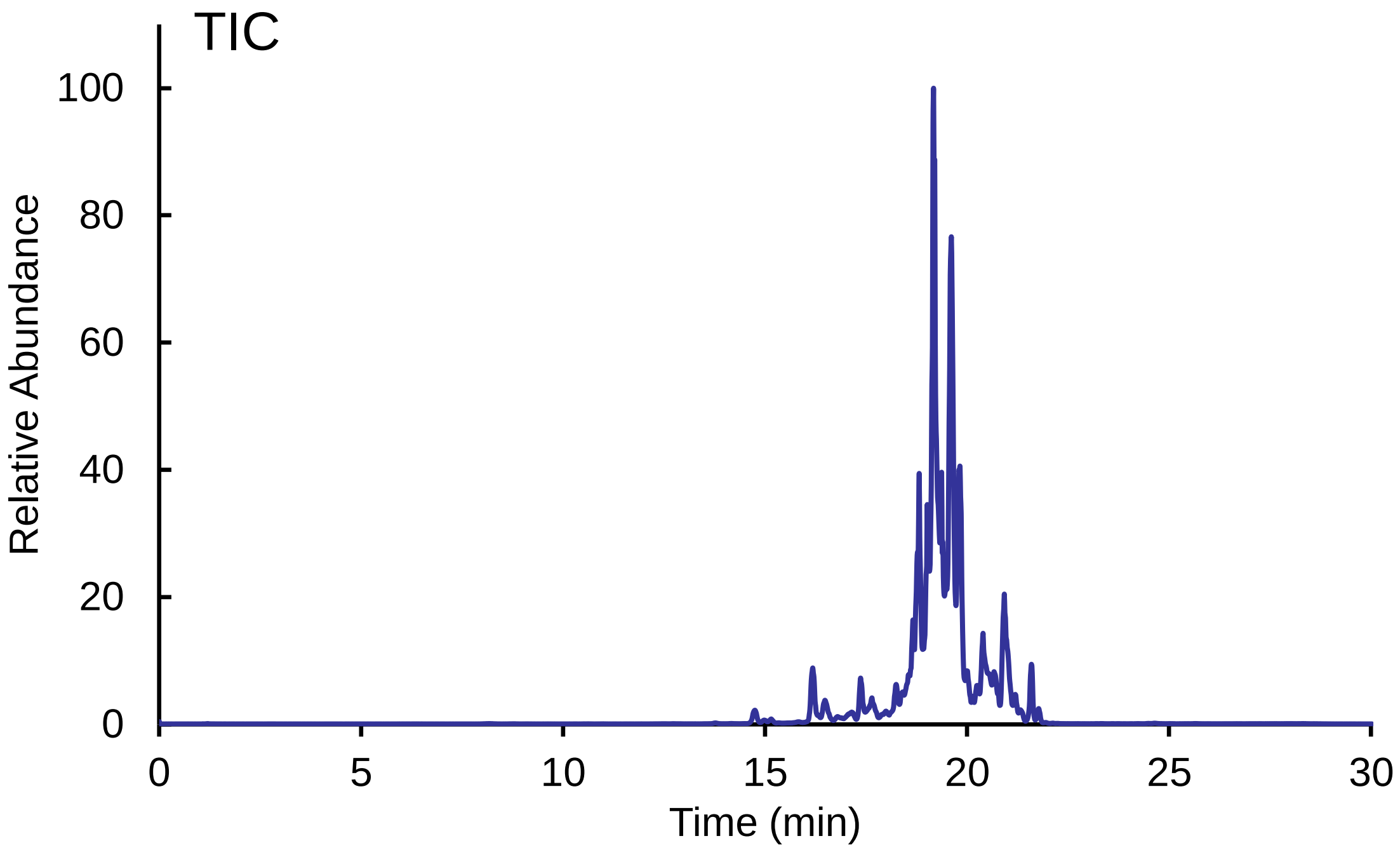
TIC
Relative Abundance
100
80
60
40
20
0
0
5
10
15
20
25
30
Time (min)

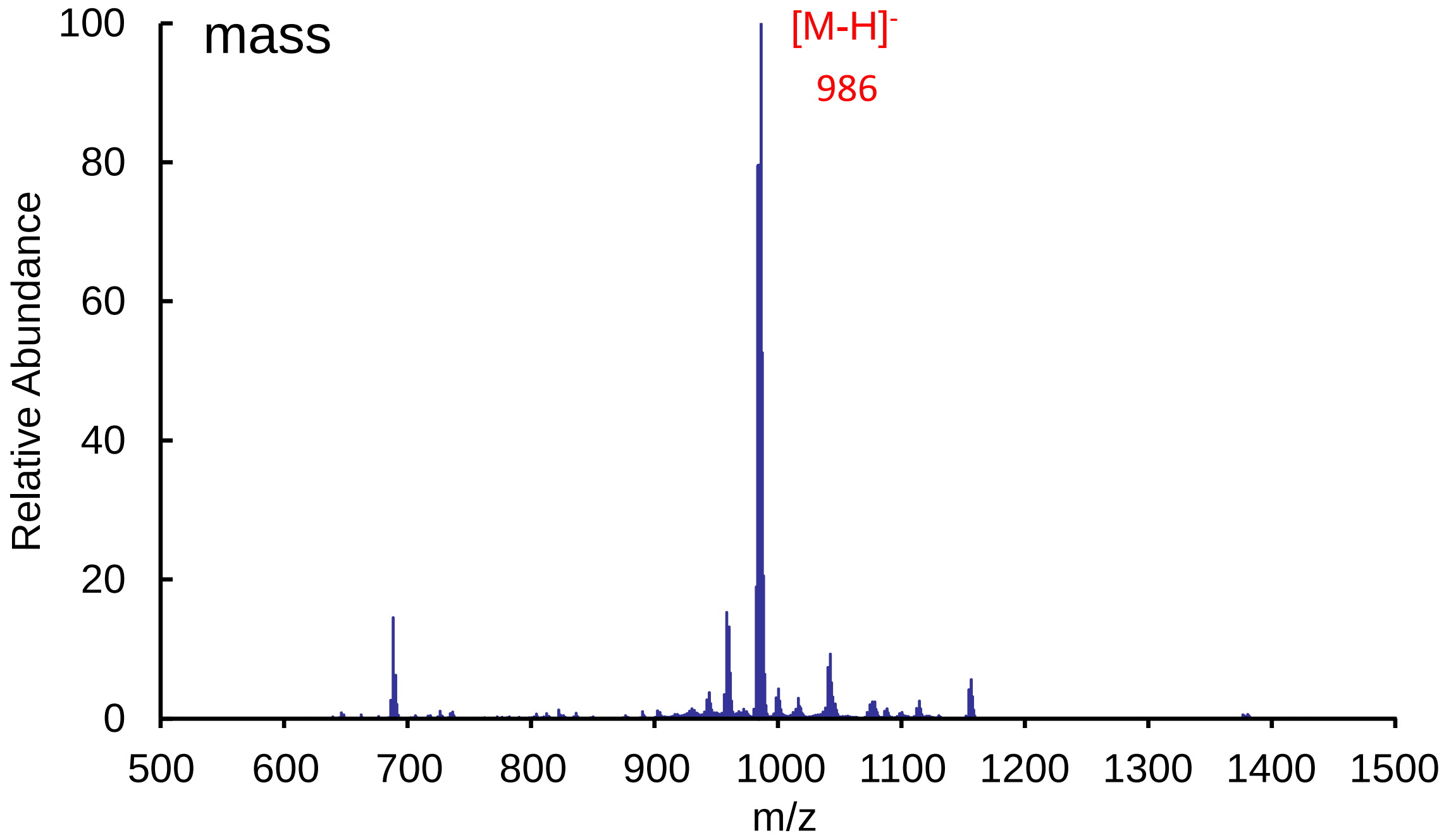
mass
[M-H]-
986
Relative Abundance
100
80
60
40
20
0
500
600
700
800
900
1000
1100
1200
1300
1400
1500
m/z

**Fig. S11**

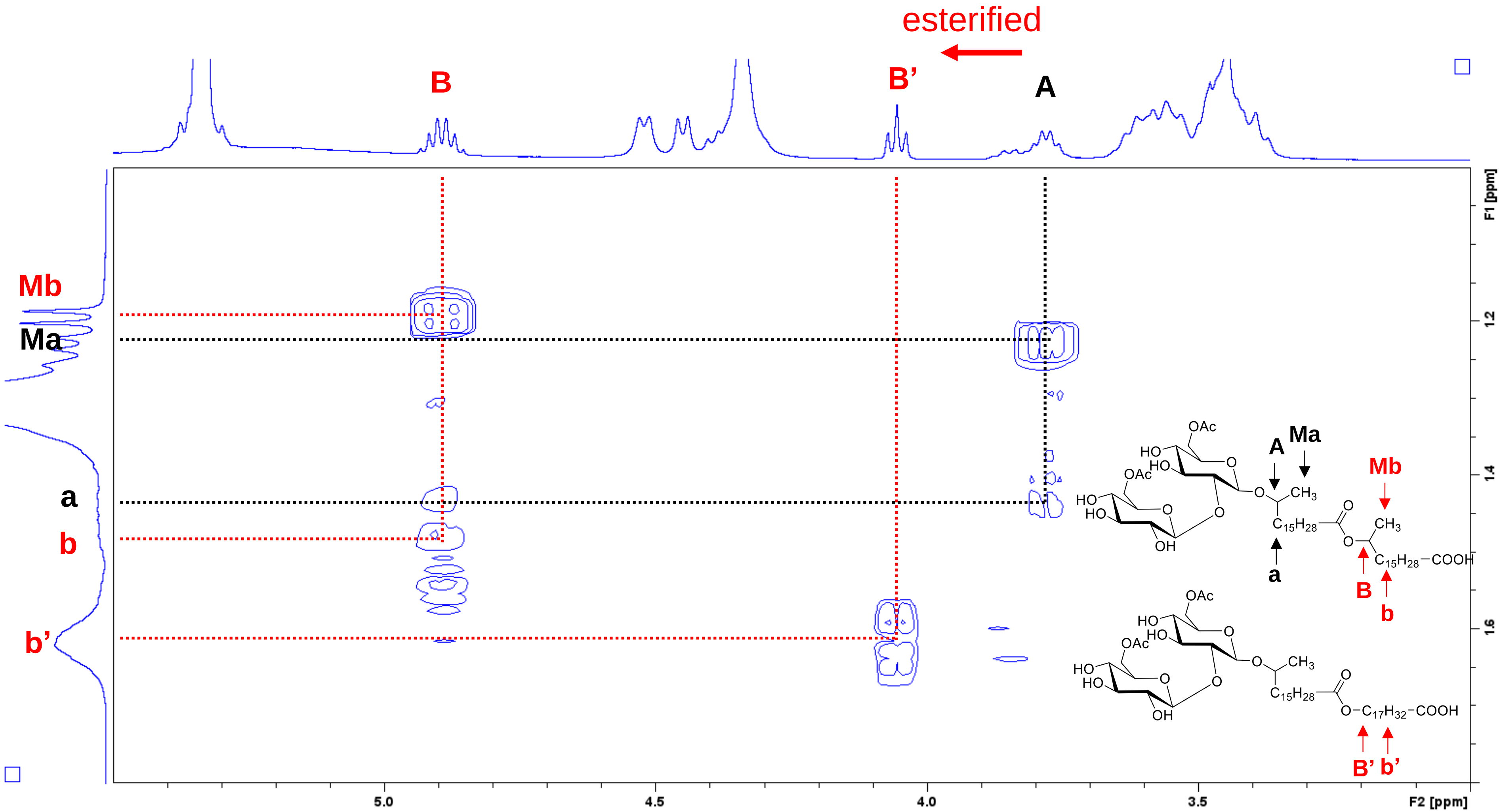

esterified
B
B'
A
Mb
Ma
a
b
b'
F1 [ppm]
1.2
1.4
1.6
5.0
4.5
4.0
3.5
F2 [ppm]
OAc
HO
HO
OAc
HO
HO
O
O
O
OH
CH3
C15H28
O
O
CH3
C15H28
COOH
C17H32
A
Ma
Mb
a
B
b
B'
b'

**Fig. S12**

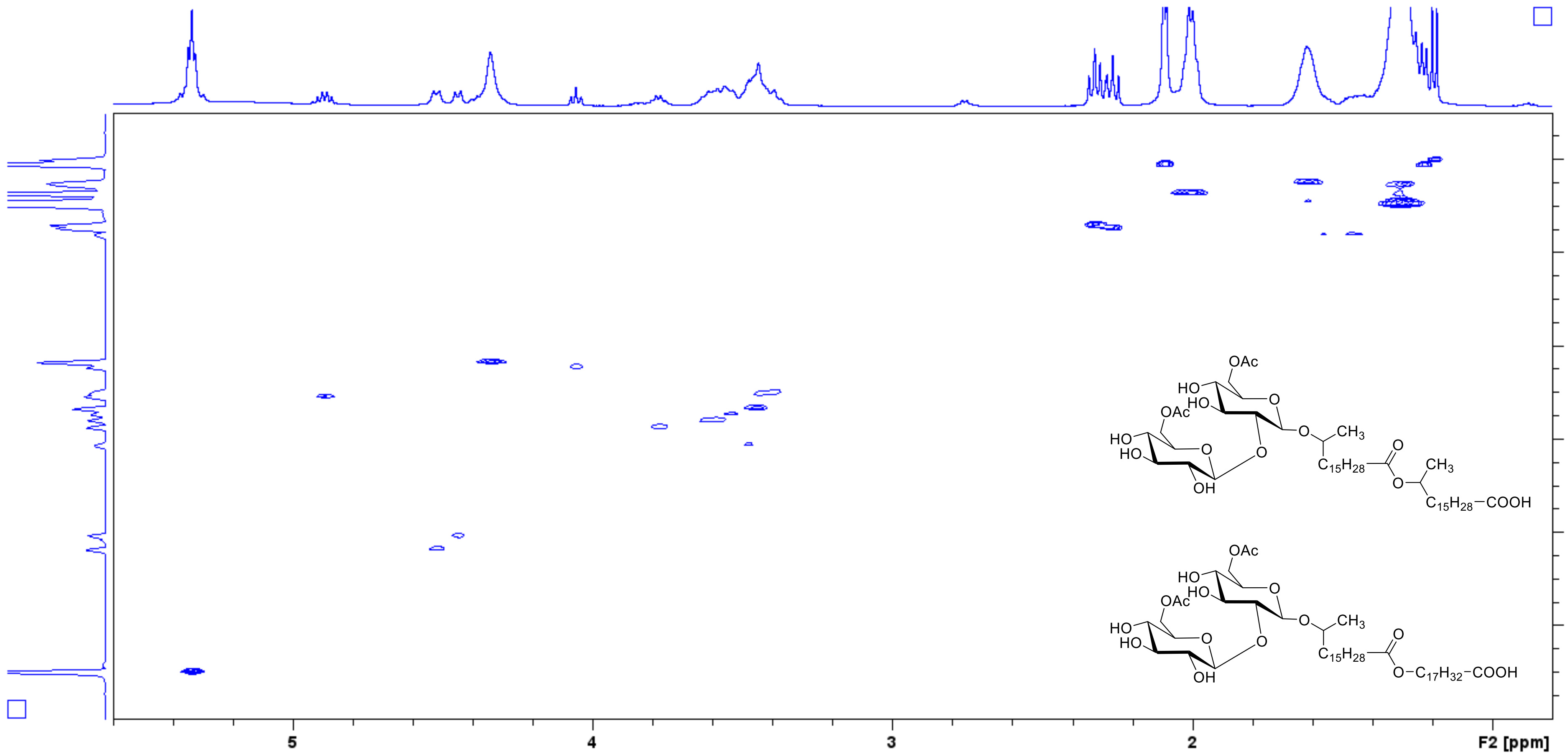
F1 [ppm]
40
60
80
100
120
5
4
3
2
F2 [ppm]
OAc
HO
HO
OAc
O
O
CH3
HO
HO
O
O
OH
C15H28
O
O
CH3
C15H28
COOH
O
O-C17H32-COOH

**Fig. S13**

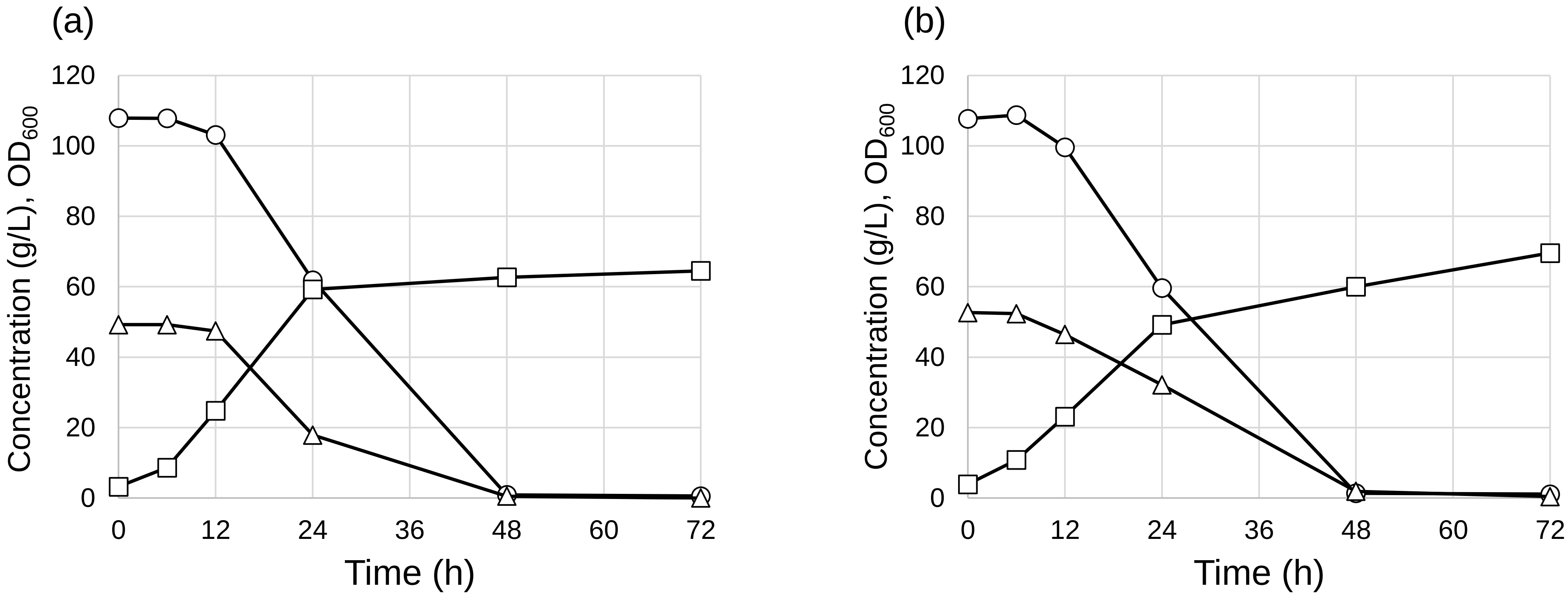


(a) rapeseed oil, (b) rice oil, circle: glucose, triangle: oil, square: $OD_{600}$

**Measurement of glucose**

The sampled medium was centrifuged to remove oil and precipitate, and the aqueous phase was diluted with 5 mM sulfuric acid for glucose concentration measurements by HPLC. The Aminex HPX-87 H column (Bio-Rad Laboratories, Hercules, CA, USA) with a guard column (Cation H Cartridges 30 × 4.6 mm; Bio-Rad) was used under the following conditions: the column oven temperature of at 65°C, 5 mM $H_2SO_4$ was used as the mobile phase buffer, and flow rate of 0.6 mL/min.

**Measurements of oil and cell growth**

Hexane was added to the sampled medium. The mixture was stirred vigorously and then centrifuged to separate the hexane and aqueous phases. The hexane phase was collected and dried under reduced pressure, and the remaining oil was weighed. Next, methanol was added to the aqueous phase, the mixture was stirred vigorously, and the yeast cells were precipitated by centrifugation. After washing with water once, the turbidity ($OD_{600}$) was measured.
