# Supplementary material for: Updated component analysis method for naturally occurring sophorolipids from Starmerella bombicola
Source: Appl Microbiol Biotechnol. 2024 Apr 12;108(1):296. doi: 10.1007/s00253-024-13138-x (PMC11009742; doi:10.1007/s00253-024-13138-x)
Supplement: Supplementary file 1 — Supplementary file1 (PDF 766 KB) [file 253_2024_13138_MOESM1_ESM.pdf]

Supplementary Information (SI)

Applied Microbiology and Biotechnology

Updated component analysis method for naturally occurring sophorolipids from *Starmerella bombicola*

Yosuke Kobayashi[1], Qiushi Li[1], Kazunori Ushimaru[2], Makoto Hirota[1], Tomotake Morita[2], Tokuma Fukuoka[2]*

[1] Allied Carbon Solutions Co., Ltd., 847-1 Ozuwa, Numazu, Shizuoka 410-0873, Japan

[2] Research Institute for Sustainable Chemistry, National Institute of Advanced Industrial Science and Technology (AIST), Tsukuba Central 5-2, 1-1-1, Higashi, Tsukuba, Ibaraki 305-8565, Japan

*Corresponding author

(TEL: +81-50-3521-1265; E-mail: t-fukuoka@aist.go.jp)

ORCID: 0000-0003-1814-7046

Table of contents

Supplementary Tables

Table S1 NMR data for the compound X (methanol-*d4*, 400 MHz)

Table S2 NMR data for the compound Y (methanol-*d4*, 400 MHz)

Supplementary Figures

Fig. S1 The numbered chemical structures of compound X, Y, and Z

Fig. S2 LC-MS analysis of compound X, isolated from *S. bombicola*

Total ion chromatogram (TIC) and mass spectrum

Fig. S3 The 400 MHz ^{1}H-NMR spectrum of compound X, isolated from *S. bombicola*

Fig. S4 Partial ^{1}H-^{1}H COSY spectrum of compound X, isolated from *S. bombicola*

F1 axis: ^{1}H-NMR spectrum ranging from 3.0 to 6.0 ppm; F2 axis: ^{1}H-NMR spectrum ranging from 3.0 to 6.0 ppm

Fig. S5 HSQC spectrum of compound X, isolated from *S. bombicola*

F1 axis: ^{13}C-NMR spectrum ranging from 10 to 140 ppm; F2 axis: ^{1}H-NMR spectrum ranging from 0.8 to 6.0 ppm

Fig. S6 LC-MS analysis of compound Y, isolated from *S. bombicola*.

Fig. S7 The 400 MHz ^{1}H-NMR spectrum of compound Y, isolated from *S. bombicola*

Fig. S8 Partial ^{1}H-^{1}H COSY spectrum of compound Y, isolated from *S. bombicola*

F1 axis: ^{1}H-NMR spectrum ranging from 3.0 to 4.8 ppm; F2 axis: ^{1}H-NMR spectrum ranging from 3.0 to 4.8 ppm

Fig. S9 HSQC spectrum of compound Y, isolated from *S. bombicola*

F1 axis: ^{13}C-NMR spectrum ranging from 10 to 140 ppm; F2 axis: ^{1}H-NMR spectrum ranging from 0.8 to 5.6 ppm

Fig. S10 LC-MS analysis of compound Z, isolated from *S. bombicola*.

Fig. S11 Partial ^{1}H-^{1}H COSY spectrum of compound Z, isolated from *S. bombicola*

F1 axis: ^{1}H-NMR spectrum ranging from 1.0 to 1.8 ppm; F2 axis: ^{1}H-NMR spectrum ranging from 3.0 to 5.5 ppm

Fig. S12 HSQC spectrum of compound Z, isolated from *S. bombicola*

F1 axis: ^{13}C-NMR spectrum ranging from 10 to 140 ppm; F2 axis: ^{1}H-NMR spectrum ranging from 0.8 to 5.6 ppm

Fig. S13 Culture profiles of jar fermentation of *S. bombicola*

(a) Rapeseed oil, (b) rice bran oil; circle: glucose; triangle: oil; square: OD_{600}

Table S1 NMR data for the compound X (methanol-*d4*, 400 MHz)

	^{13}C-NMR δ (ppm)		^{1}H-NMR δ (ppm)	*J* (Hz)
**Saccharides (G1~4)**				
C-1	92.6 (G3),<br>101.2 (G1),<br>104.2 (G2,4)	H-1	4.48 d (G1),<br>4.59 d (G2,4),<br>5.63 d (G3)	7.7
C-2	74.6 (G2,4),<br>81.1 (G3),<br>82.4 (G1),	H-2	3.58 t (G3),<br>3.20-3.65 m	9.0
C-3	76.1–76.8	H-3	3.20–3.65 m	
C-4	69.3–70.0	H-4	3.20–3.65 m	
C-5	73.5–74.2	H-5	3.20–3.65 m	
C-6	60.8–61.3	H-6	3.70, 3.85 m	
C-6 (acetylated)	63.4–63.6	H-6 (acetylated)	4.22, 4.39 m	
**Acetyl groups**				
–C=O (C-6)	171.6			
$–CH_3$ (C-6)	19.4, 19.6		2.07, 2.09 s	
**Acyl group**				
–C=O	173.7			
$–CO\text{-}\underline{CH_2}–$	33.5		2.44 m	
$–CO\text{-}CH_2\underline{CH_2}–$	24.2		1.64 b	
$–(CH_2)_n–$	24.8–29.6		1.3–1.4 b	
$–CH{=}CH\text{-}\underline{CH_2}–$	26.8		2.0–2.1 b	
–CH=CH–	129.5		5.37 m	
$–OCH(CH_3)\text{-}\underline{CH_2}–$	36.5		1.64 b	
$–O\underline{CH}(CH_3)–$	77.3		3.74 m	
$–CH_3$	20.5		1.22 d	6.2

Table S2 NMR data for the compound Y (methanol-*d4*, 400 MHz)

	^{13}C-NMR δ (ppm)		^{1}H-NMR δ (ppm)	*J* (Hz)
**Saccharides**				
C-1'	101.2	H-1'	4.47 d	7.7
C-2'	82.4	H-2'	3.2–3.5 m	
C-3'	76.1 or 76.4	H-3'	3.2–3.5 m	
C-4'	70.0 or 70.1	H-4'	3.2–3.5 m	
C-5'	73.5 or 74.2	H-5'	3.2–3.5 m	
C-6'	63.4 or 63.5	H-6'a	4.21 m	
		H-6'b	4.37 m	
C-1''	104.3	H-1''	4.57 d	7.8
C-2''	74.7	H-2''	3.2–3.5 m	
C-3''	76.1 or 76.4	H-3''	3.2–3.5 m	
C-4''	70.0 or 70.1	H-4''	3.2–3.5 m	
C-5''	73.5 or 74.2	H-5''	3.2–3.5 m	
C-6''	63.4 or 63.5	H-6''a	4.21 m	
		H-6''b	4.37 m	
**Acetyl groups**				
–C=O (C-6',6'')	171.3			
$–CH_3$ (C-6',6'')	19.4, 19.5		2.06, 2.08 s	
**Glycerol groups**				
$–CH_2O–$	65.1		4.08, 4.15 m	
–CHO–	69.7		3.85 m	
$–CH_2OH$	62.7		3.57 m	
**Acyl group**				
–C=O	174.2			
–CO-$\underline{CH_2}$–	33.6		2.37 t	7.4
–CO-$CH_2\underline{CH_2}$–	24.6		1.62 b	
$–(CH_2)_n–$	24.7–29.6		1.2–1.4 b	
–CH=CH-$\underline{CH_2}$–	26.8		2.0–2.1 b	
–CH=CH–	129.5		5.36 m	
$–OCH(CH_3)$-$\underline{CH_2}$–	36.5		1.62 b	
–O$\underline{CH}(CH_3)$–	77.2		3.76 m	
$–CH_3$	20.5		1.21 d	6.2

Fig. S1

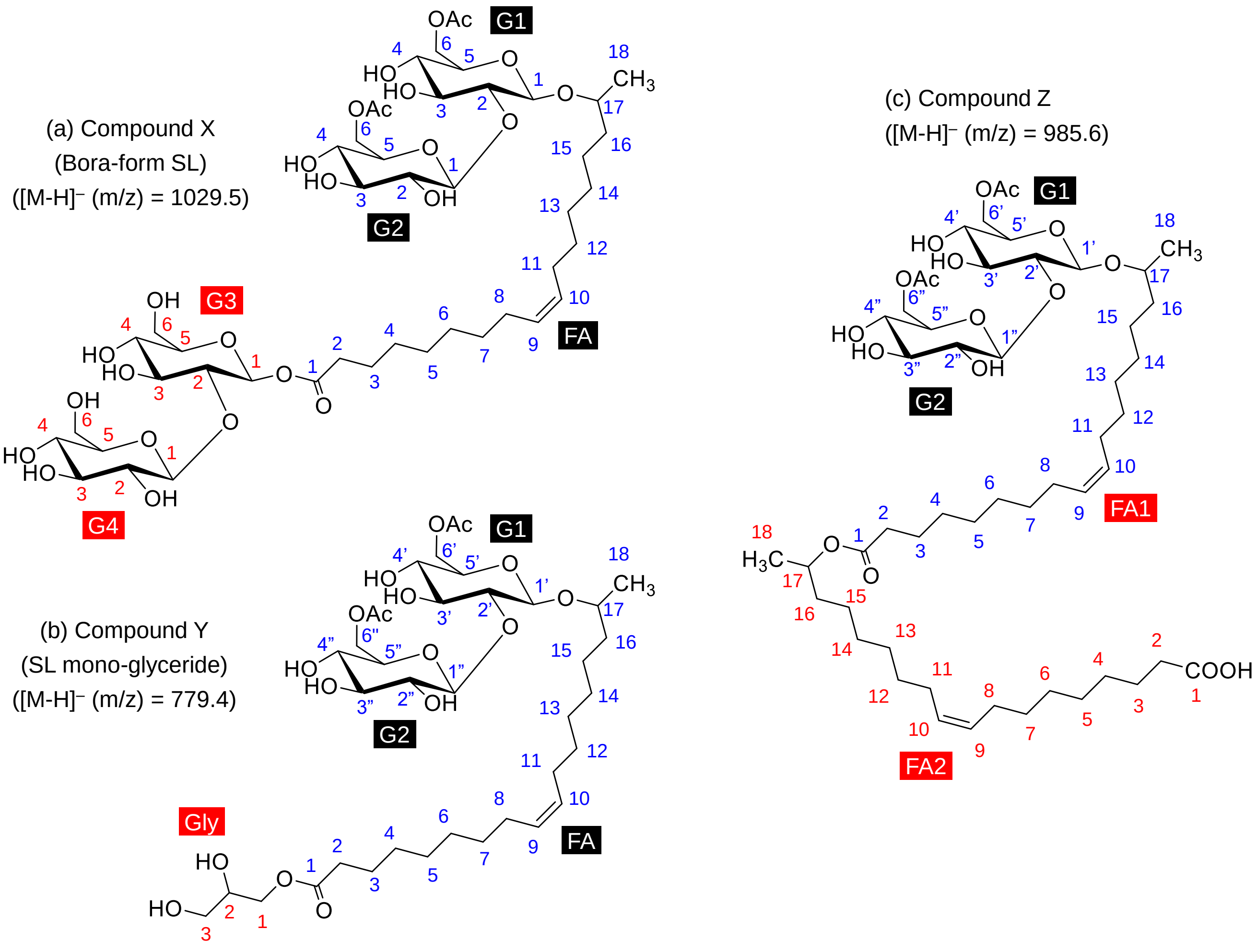
(a) Compound X
(Bora-form SL)
([M-H]⁻ (m/z) = 1029.5)
G1
G2
G3
G4
FA
(b) Compound Y
(SL mono-glyceride)
([M-H]⁻ (m/z) = 779.4)
G1
G2
Gly
FA
(c) Compound Z
([M-H]⁻ (m/z) = 985.6)
G1
G2
FA1
FA2

Fig. S2

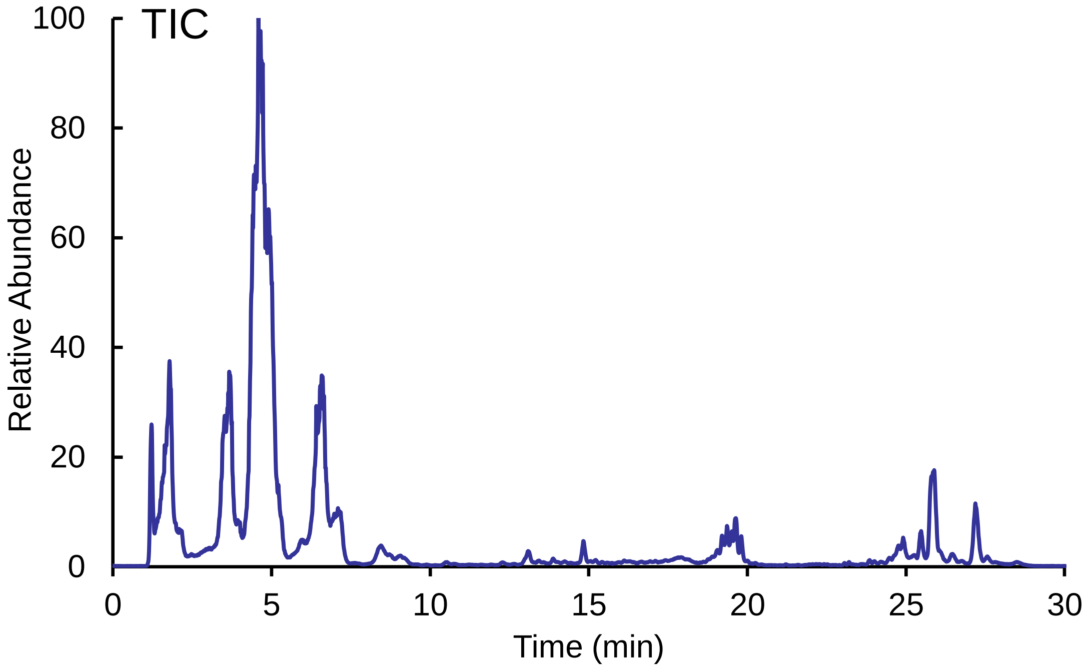

TIC
Relative Abundance
100
80
60
40
20
0
0
5
10
15
20
25
30
Time (min)

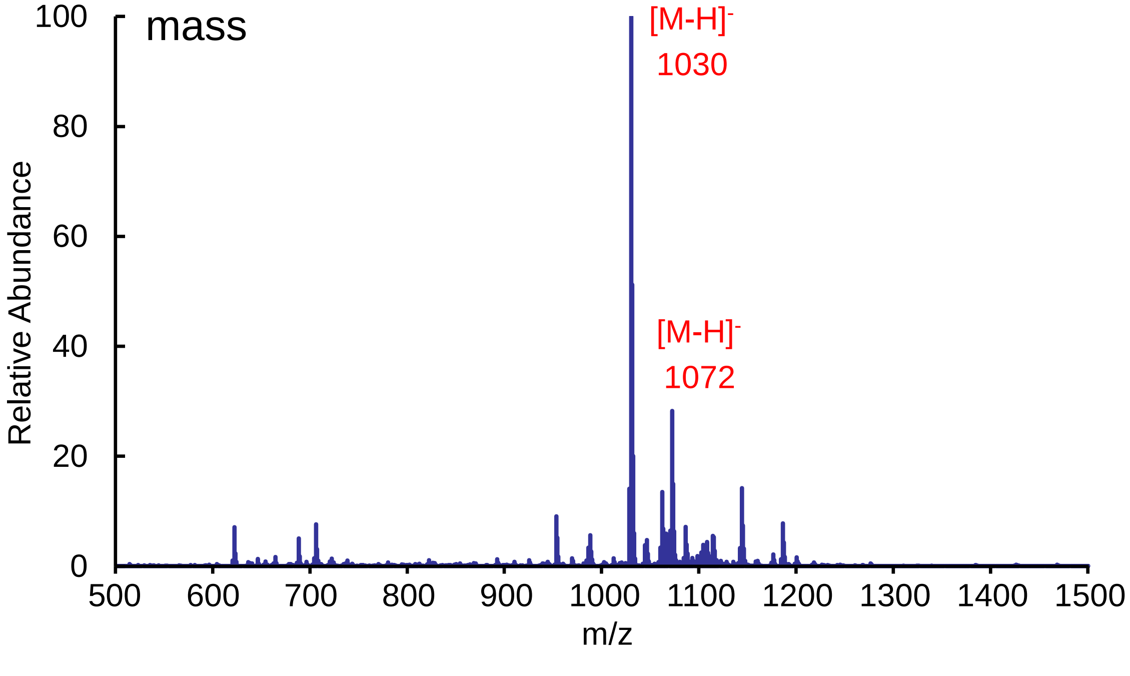

mass
Relative Abundance
100
80
60
40
20
0
500
600
700
800
900
1000
1100
1200
1300
1400
1500
m/z
[M-H]-
1030
[M-H]-
1072

Fig. S3

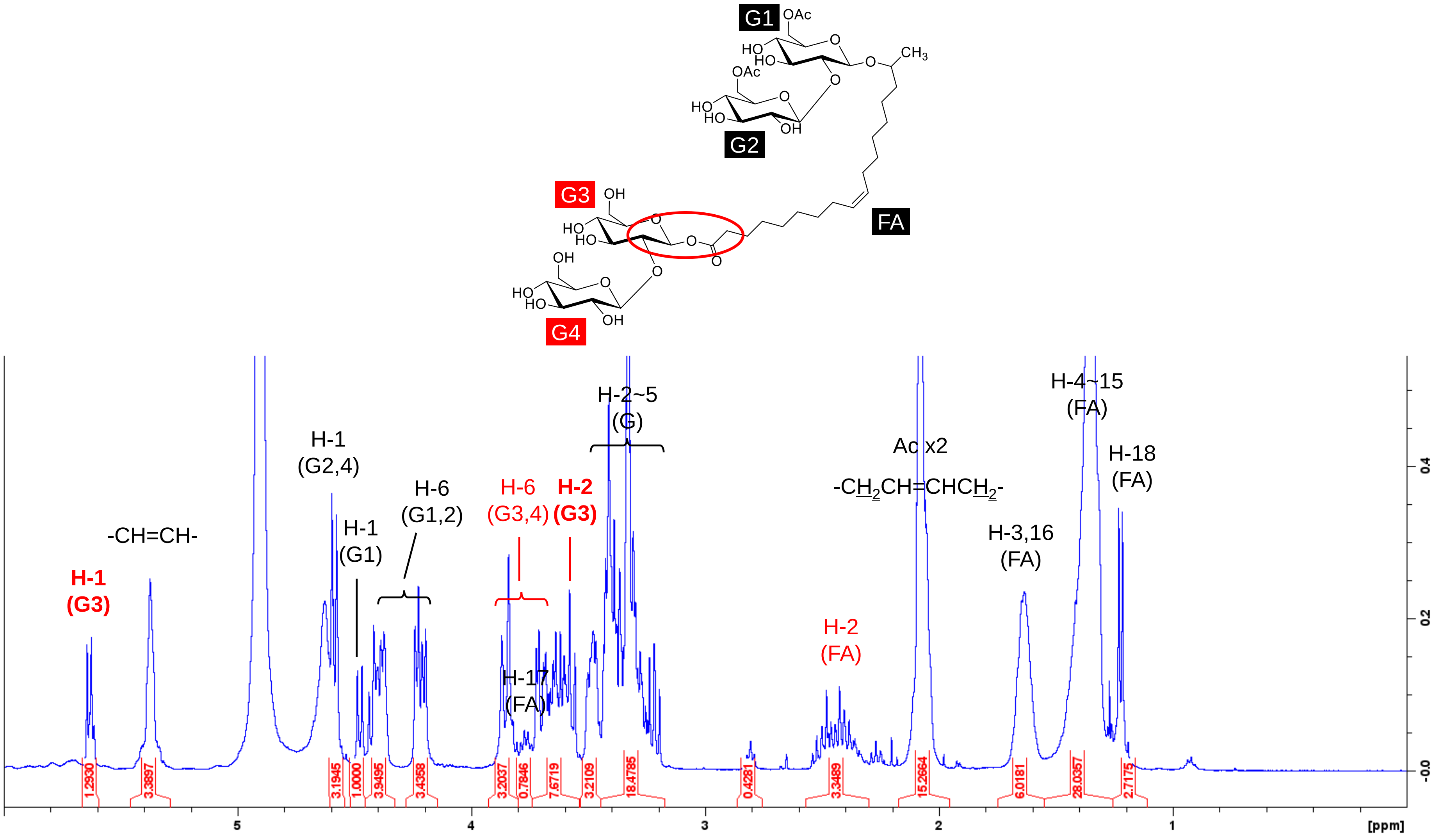
G1
OAc
HO
HO
O
O
CH3
OAc
O
HO
HO
OH
G2
G3
OH
HO
HO
O
O
O
FA
OH
O
HO
HO
OH
G4
H-1
(G3)
-CH=CH-
H-1
(G2,4)
H-1
(G1)
H-6
(G1,2)
H-6
(G3,4)
H-2
(G3)
H-17
(FA)
H-2~5
(G)
H-2
(FA)
Ac x2
-CH2CH=CHCH2-
H-3,16
(FA)
H-4~15
(FA)
H-18
(FA)
1.2930
3.3897
3.1945
1.0000
3.9495
3.4358
3.2037
0.7846
7.6719
3.2109
18.4785
0.4281
3.3489
15.2664
6.0181
28.0357
2.7175
5
4
3
2
1
[ppm]
0.4
0.2
0.0

Fig. S4

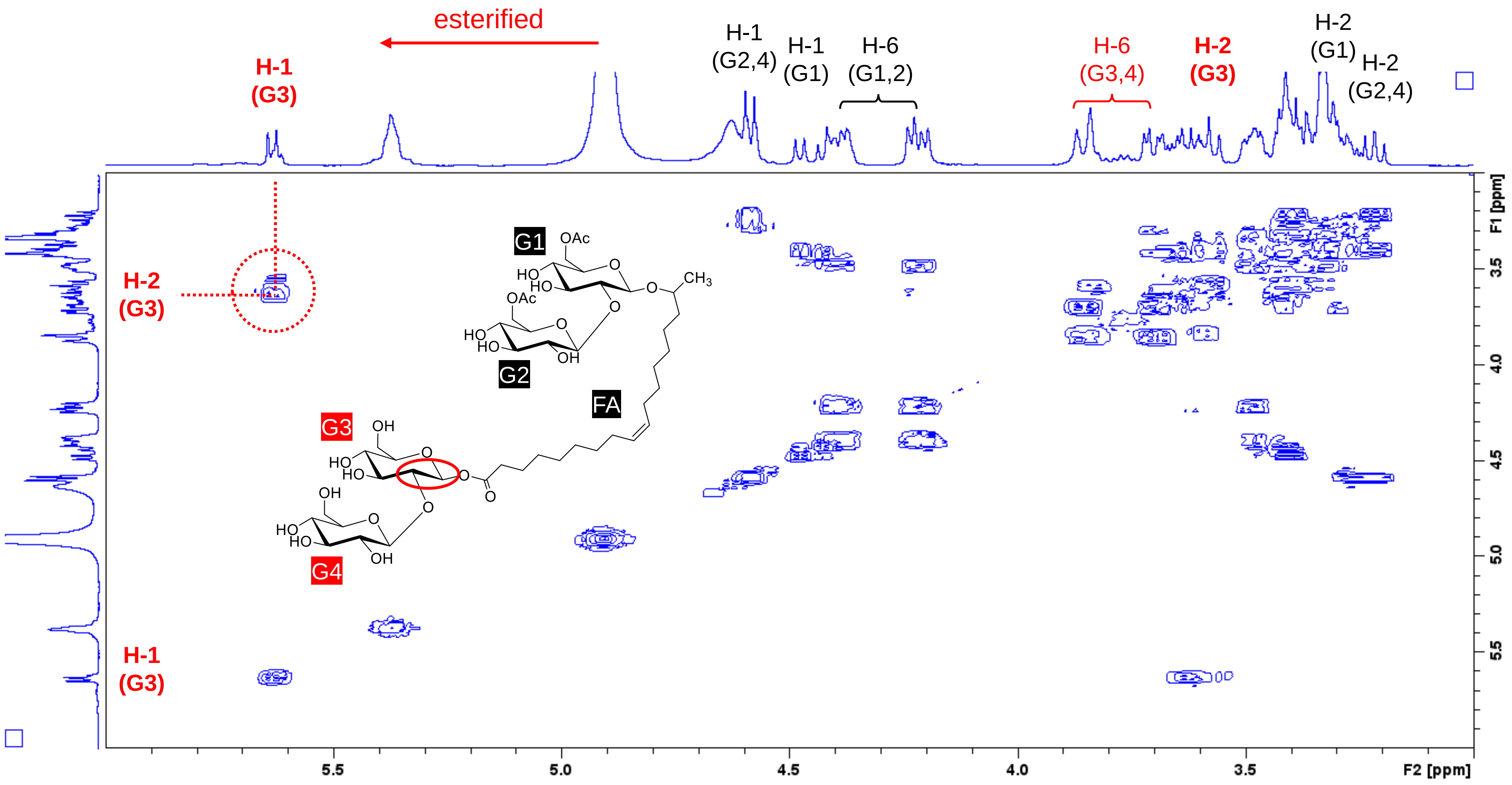

esterified
H-1
(G3)
H-1
(G2,4)
H-1
(G1)
H-6
(G1,2)
H-6
(G3,4)
H-2
(G3)
H-2
(G1)
H-2
(G2,4)
H-2
(G3)
H-1
(G3)
G1
OAc
HO
HO
OAc
CH3
HO
HO
OH
G2
FA
G3
OH
HO
HO
OH
HO
HO
OH
G4
5.5
5.0
4.5
4.0
3.5
F2 [ppm]
F1 [ppm]

Fig. S5

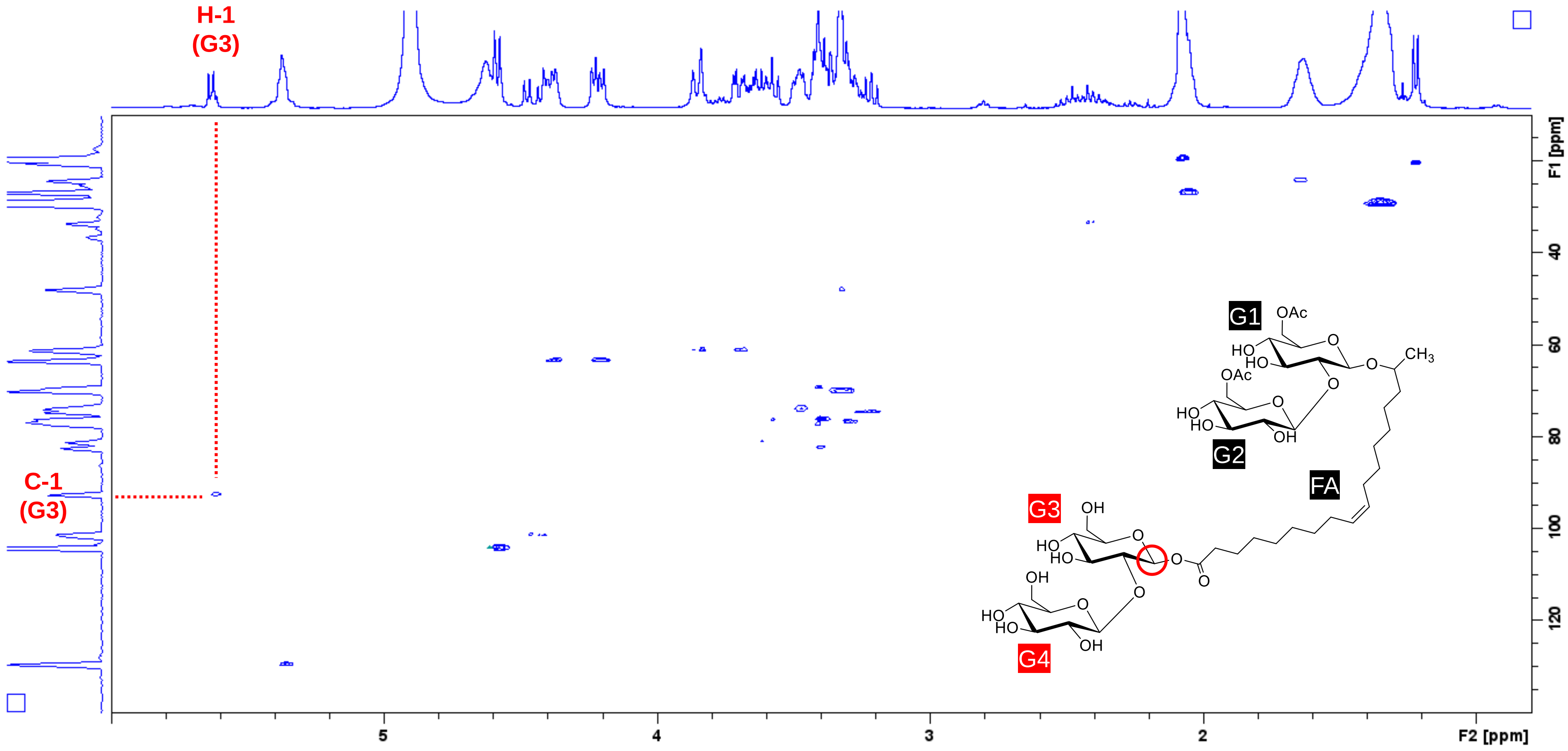

H-1
(G3)
C-1
(G3)
G1
OAc
HO
HO
O
O
CH3
OAc
O
O
HO
HO
OH
G2
FA
G3
OH
O
HO
HO
O
O
O
OH
O
HO
HO
OH
G4
5
4
3
2
F2 [ppm]
40
60
80
100
120
F1 [ppm]

Fig. S6

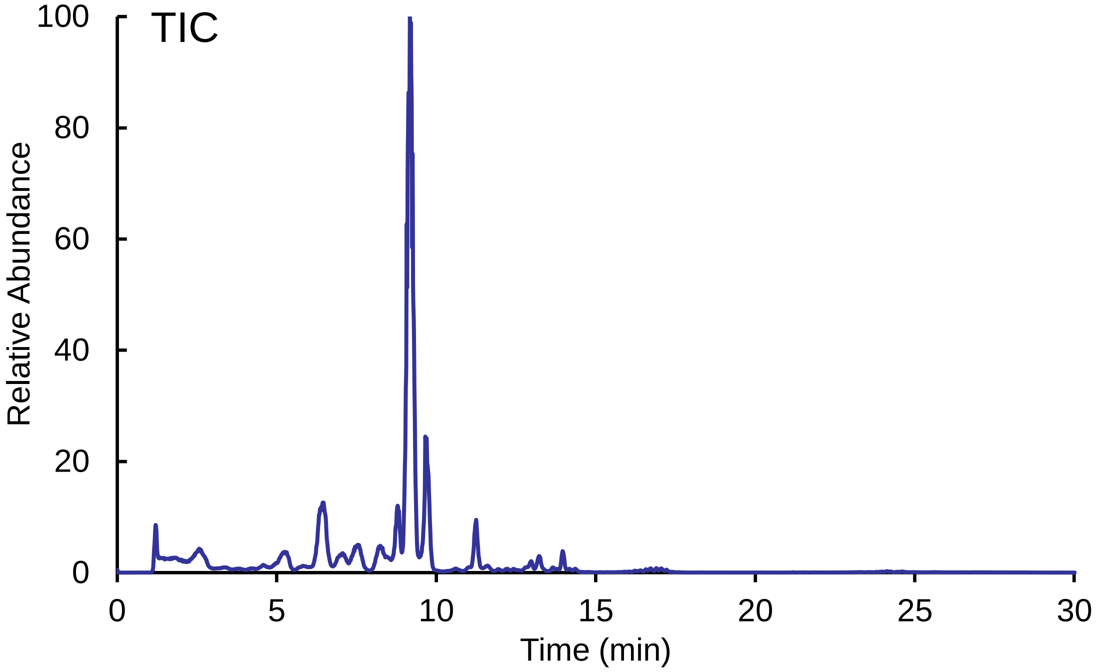
TIC
Relative Abundance
100
80
60
40
20
0
0
5
10
15
20
25
30
Time (min)

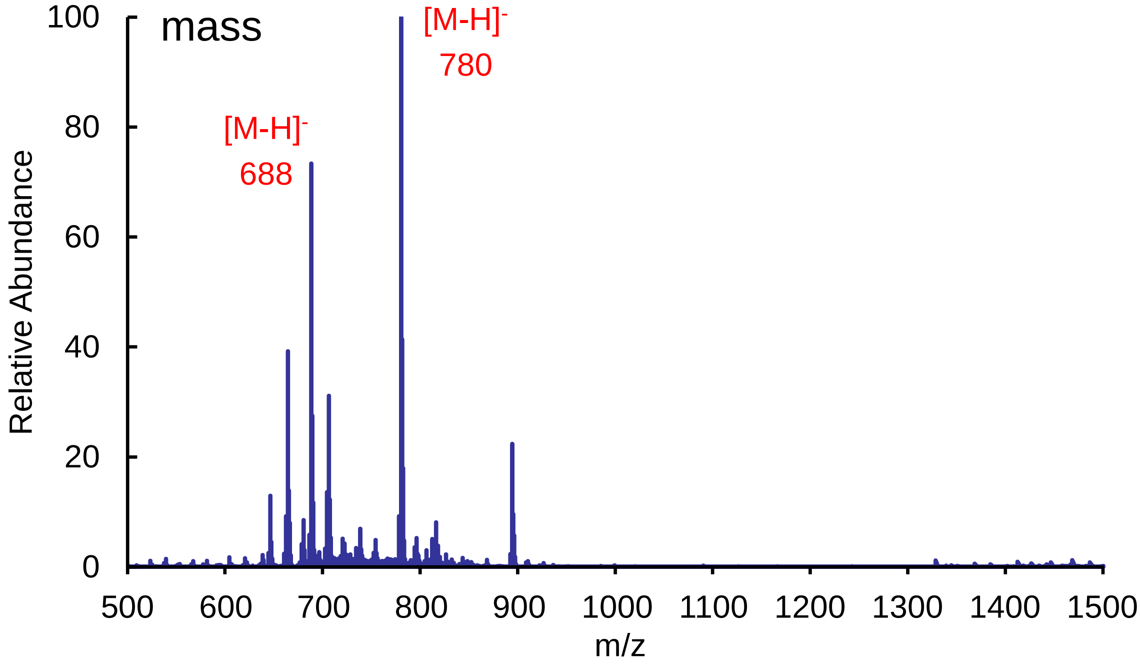
mass
Relative Abundance
[M-H]-
688
[M-H]-
780
100
80
60
40
20
0
500
600
700
800
900
1000
1100
1200
1300
1400
1500
m/z

Fig. S7

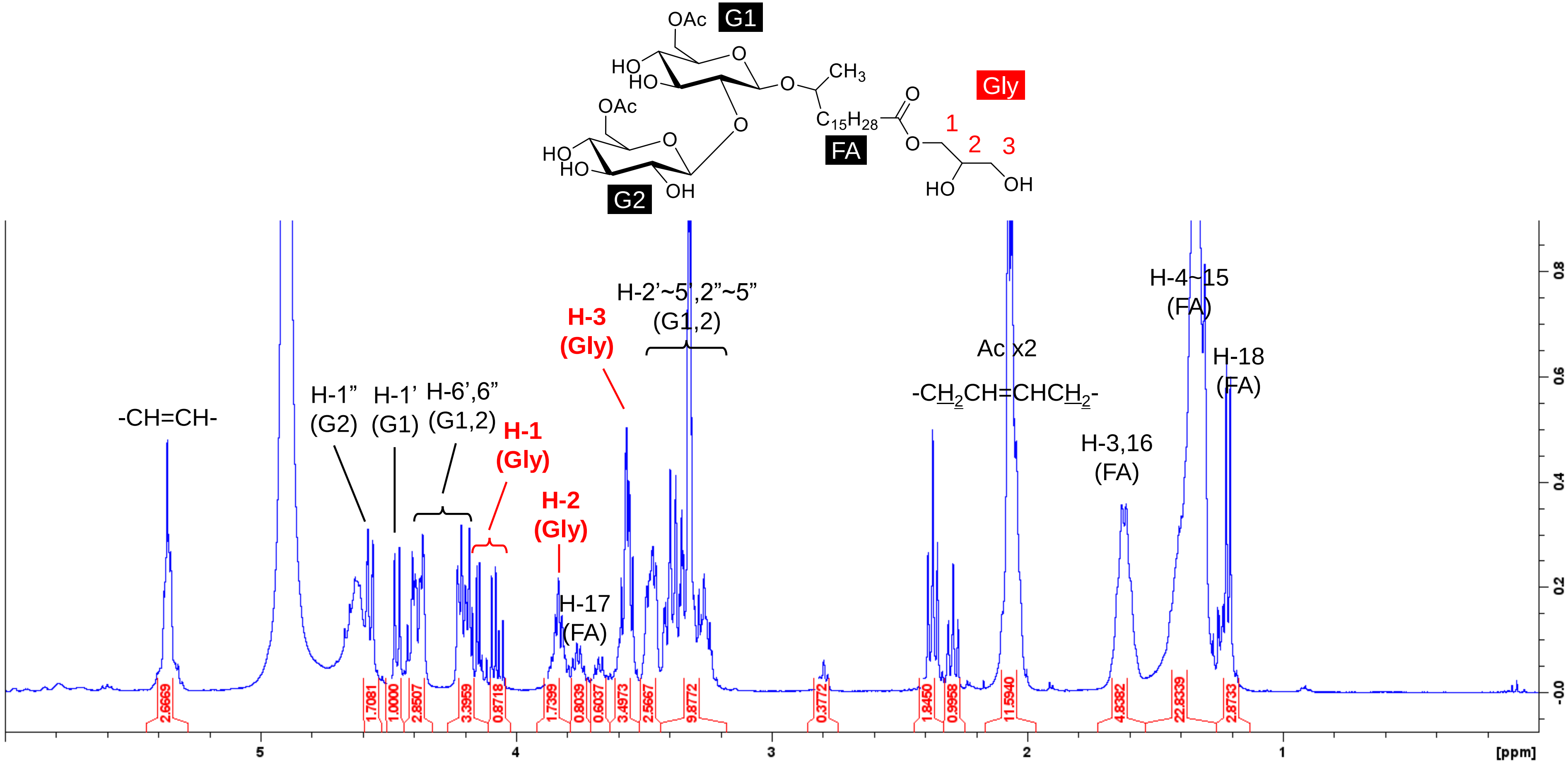

OAc
G1
HO
HO
O
O
CH_3
OAc
O
Gly
$C_{15}H_{28}$
O
1
2
3
HO
HO
O
FA
G2
OH
HO
OH
H-2'~5',2"~5"
(G1,2)
H-3
(Gly)
H-4~15
(FA)
Ac x2
H-18
(FA)
$-CH_2CH=CHCH_2-$
-CH=CH-
H-1"
(G2)
H-1'
(G1)
H-6',6"
(G1,2)
H-1
(Gly)
H-3,16
(FA)
H-2
(Gly)
H-17
(FA)
2.6669
1.7081
1.0000
2.8507
3.3959
0.8718
1.7399
0.8039
0.6037
3.4973
2.5667
9.8772
0.3772
1.8450
0.9958
11.5940
4.8382
22.8339
2.8733
5
4
3
2
1
[ppm]
0.8
0.6
0.4
0.2
0.0

Fig. S8

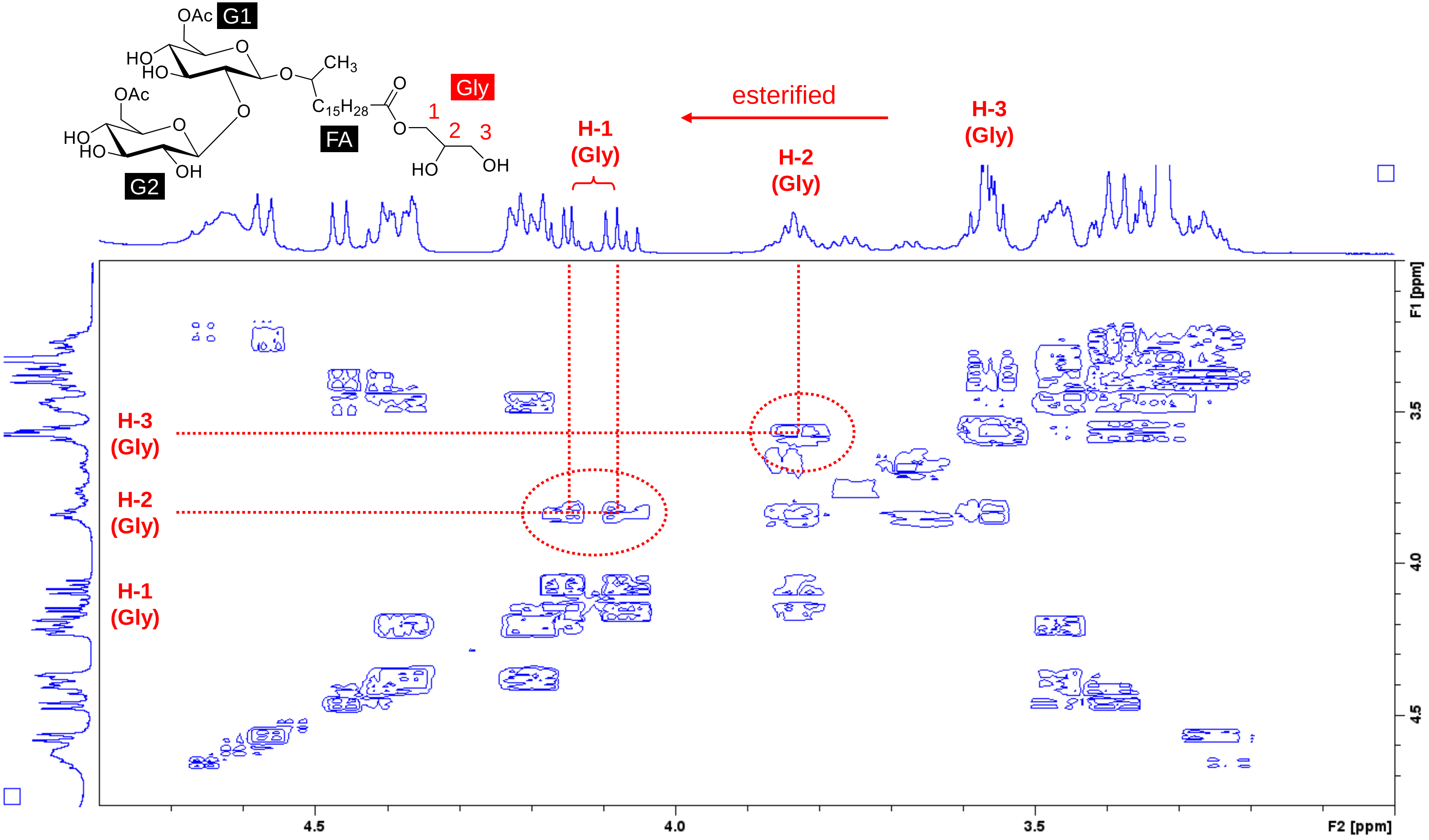
OAc
G1
HO
HO
O
O
CH3
OAc
O
C15H28
O
Gly
1
2
3
FA
HO
HO
OH
G2
HO
OH
esterified
H-1 (Gly)
H-2 (Gly)
H-3 (Gly)
H-3 (Gly)
H-2 (Gly)
H-1 (Gly)
4.5
4.0
3.5
F2 [ppm]
F1 [ppm]

Fig. S9

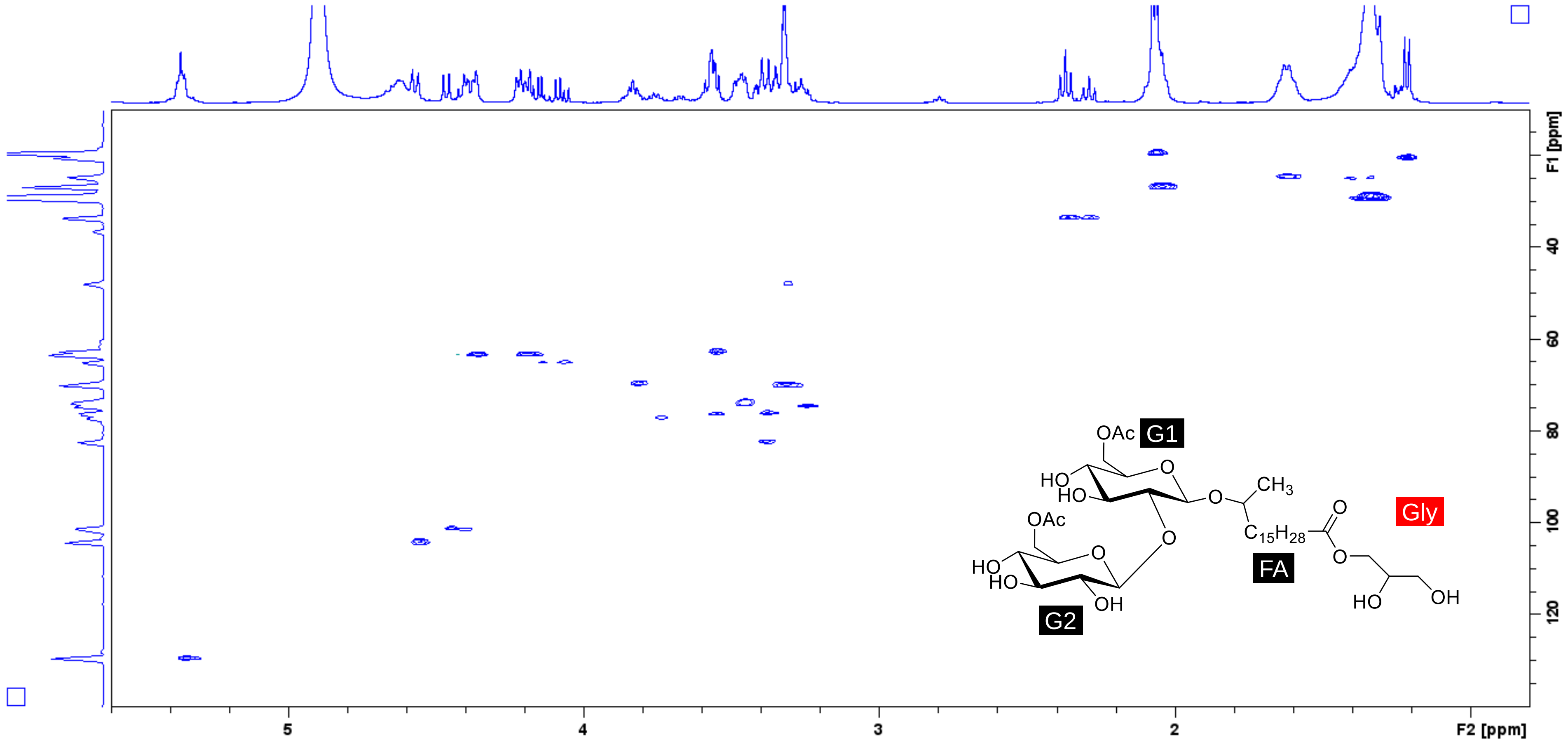

F1 [ppm]
40
60
80
100
120
5
4
3
2
F2 [ppm]
OAc
G1
HO
HO
O
O
CH3
OAc
O
O
C15H28
O
Gly
HO
HO
O
O
FA
OH
HO
OH
G2

Fig. S10

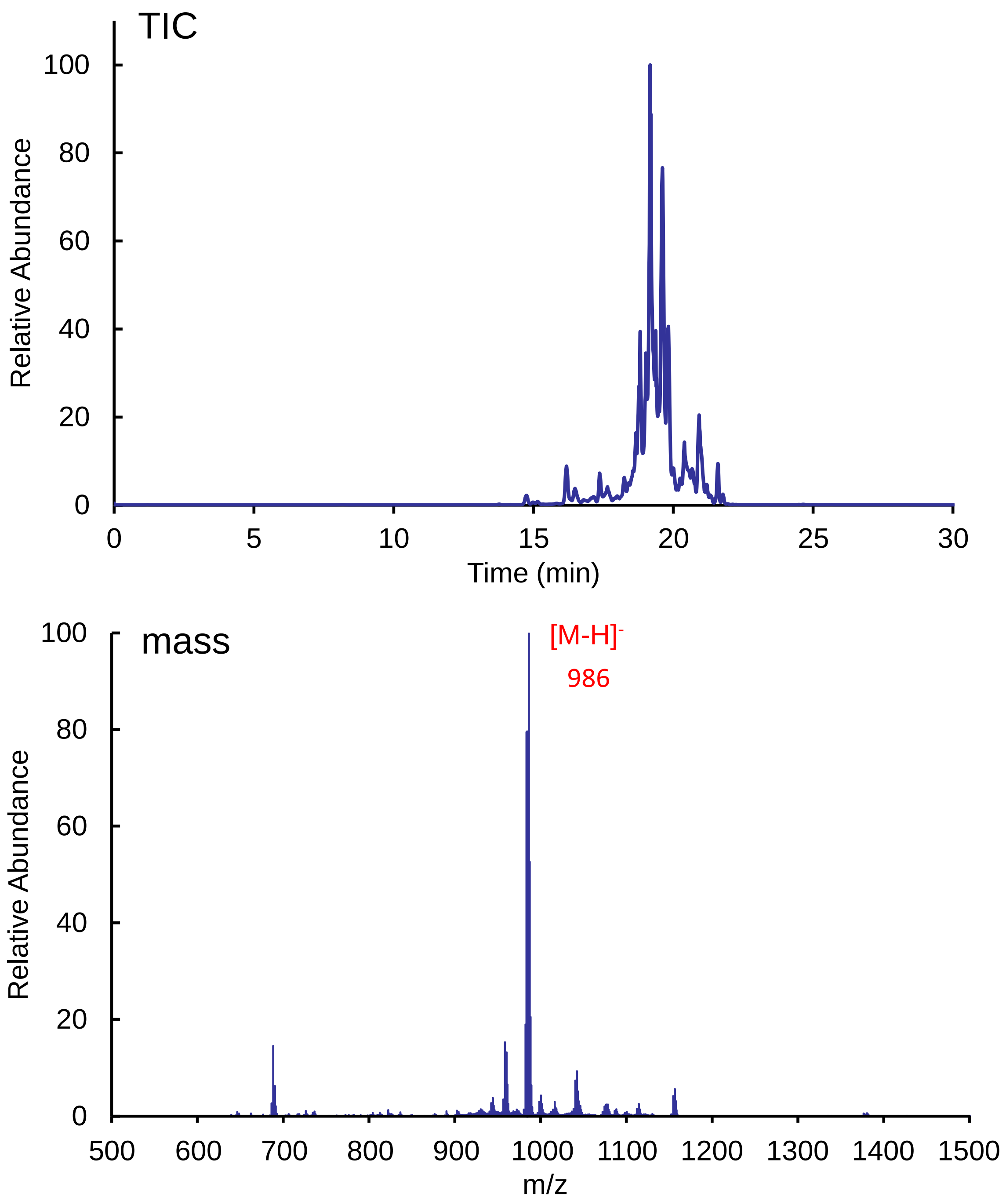

TIC
Relative Abundance
100
80
60
40
20
0
0
5
10
15
20
25
30
Time (min)
mass
[M-H]-
986
Relative Abundance
100
80
60
40
20
0
500
600
700
800
900
1000
1100
1200
1300
1400
1500
m/z

Fig. S11

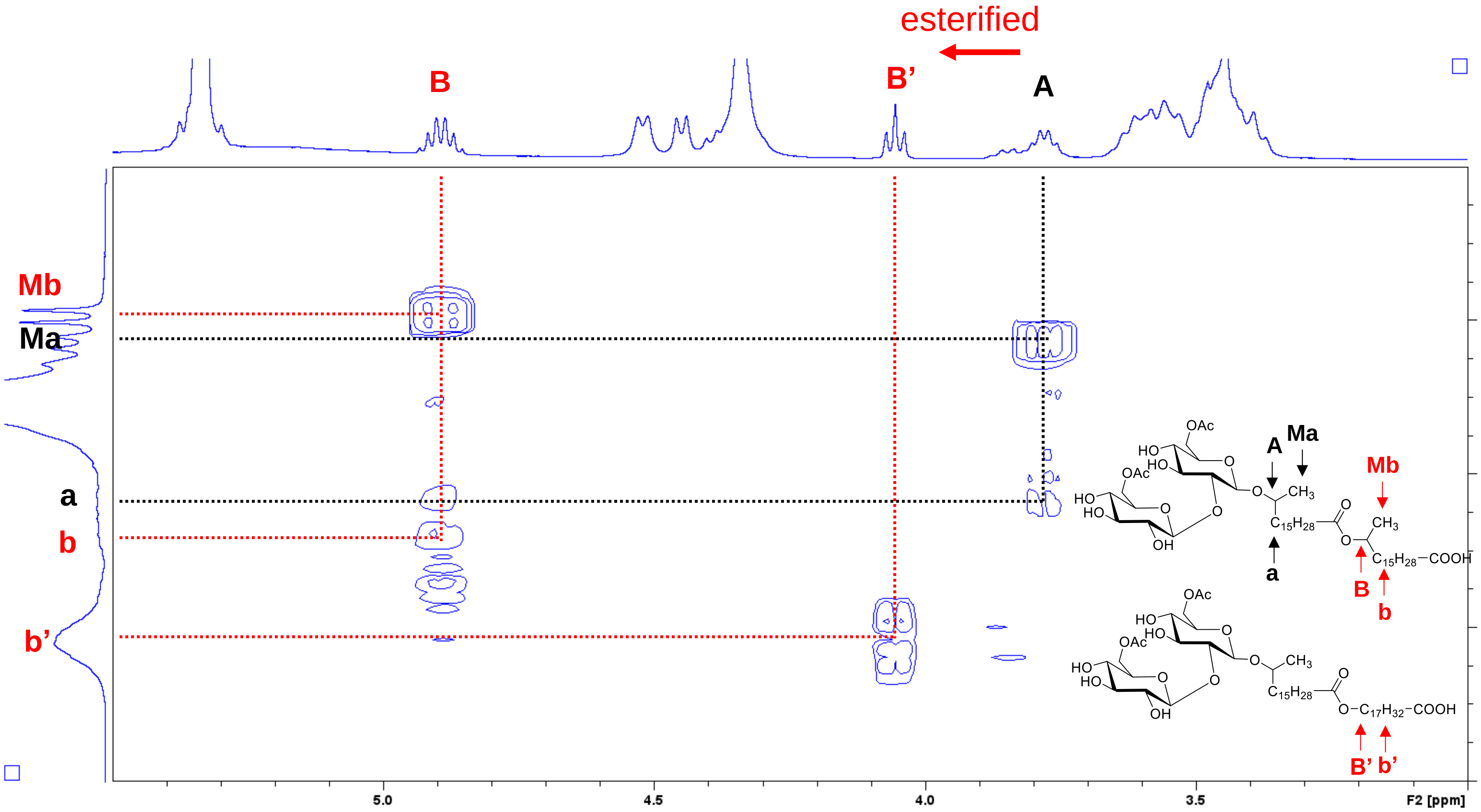

esterified
B
B'
A
Mb
Ma
a
b
b'
F1 [ppm]
1.2
1.4
1.6
5.0
4.5
4.0
3.5
F2 [ppm]
OAc
HO
HO
OAc
HO
HO
O
O
O
O
OH
A
Ma
CH3
C15H28
O
O
Mb
CH3
C15H28–COOH
a
B
b
OAc
HO
HO
OAc
HO
HO
O
O
O
O
OH
CH3
C15H28
O
O–C17H32–COOH
B' b'

Fig. S12

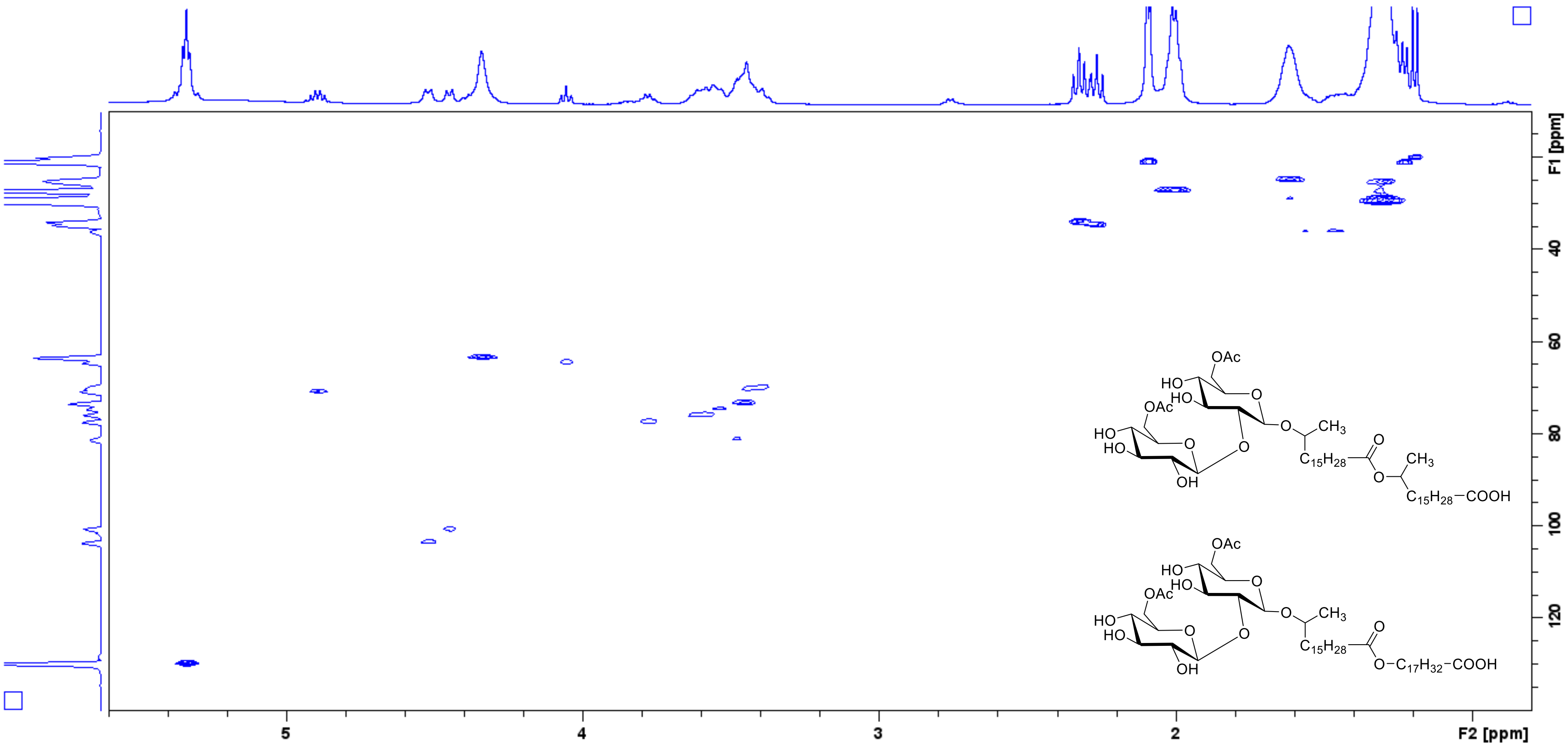

F1 [ppm]
F2 [ppm]
40
60
80
100
120
5
4
3
2
OAc
HO
HO
OAc
O
HO
HO
O
O
OH
O
CH3
C15H28
O
O
CH3
C15H28−COOH
O−C17H32−COOH

Fig. S13

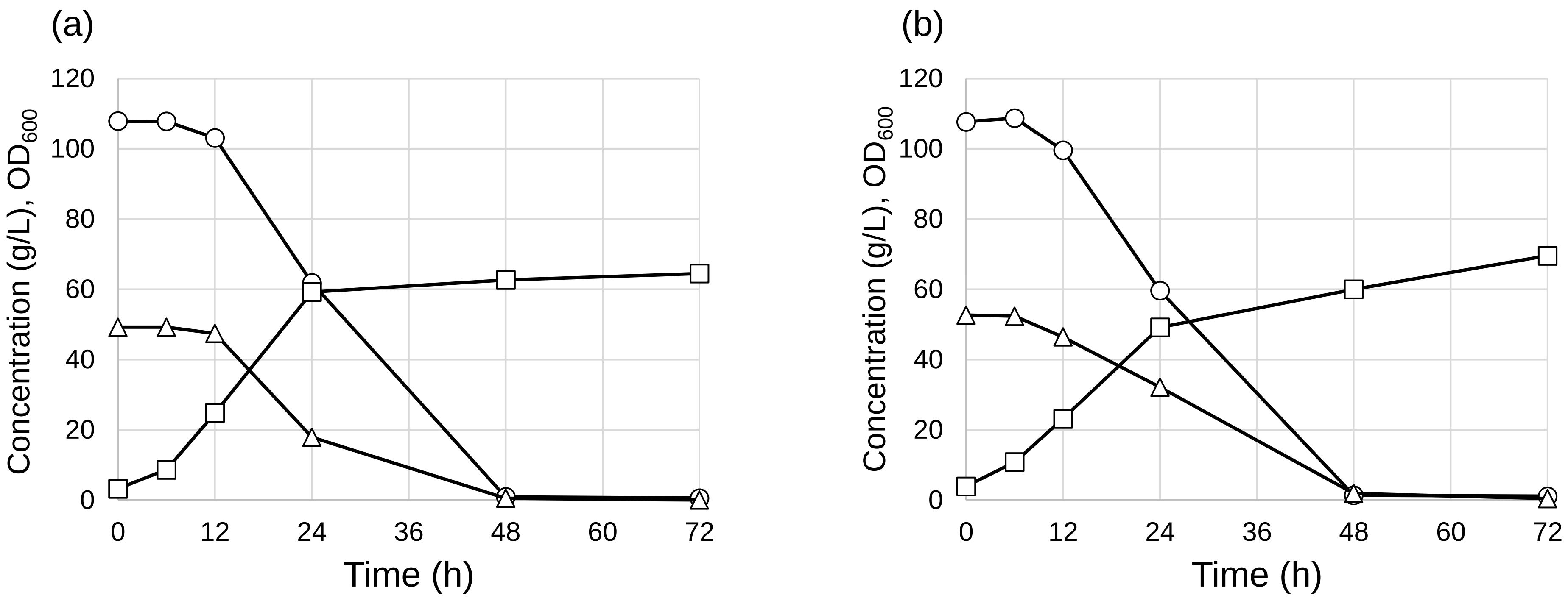

(a) rapeseed oil, (b) rice oil, circle: glucose, triangle: oil, square: OD_{600}

Measurement of glucose

The sampled medium was centrifuged to remove oil and precipitate, and the aqueous phase was diluted with 5 mM sulfuric acid for glucose concentration measurements by HPLC. The Aminex HPX-87 H column (Bio-Rad Laboratories, Hercules, CA, USA) with a guard column (Cation H Cartridges 30 × 4.6 mm; Bio-Rad) was used under the following conditions: the column oven temperature of at 65°C, 5 mM H_2SO_4 was used as the mobile phase buffer, and flow rate of 0.6 mL/min.

Measurements of oil and cell growth

Hexane was added to the sampled medium. The mixture was stirred vigorously and then centrifuged to separate the hexane and aqueous phases. The hexane phase was collected and dried under reduced pressure, and the remaining oil was weighed. Next, methanol was added to the aqueous phase, the mixture was stirred vigorously, and the yeast cells were precipitated by centrifugation. After washing with water once, the turbidity (OD_{600}) was measured.
